# Sex- and country-specific associations of hyperuricemia and inflammation with vascular aging across populations with diverse cardiovascular risks

**DOI:** 10.3389/fmed.2025.1737935

**Published:** 2026-01-21

**Authors:** Agnė Laučytė-Cibulskienė, Jolita Badarienė, José-Antonio Costa-Muñoz, Christopher Nilsson, Sigita Glaveckaitė, Anders Christensson, Luis Salar-Ibáñez, Egidija Rinkūnienė, Gunnar Engström, Ieva Berankytė, José Chordá-Ribelles, Karl-Heinz Herzig, Enrique Rodilla

**Affiliations:** 1 Department of Clinical Sciences Malmo, Lund University, Malmö, Sweden; 2 Clinic of Cardiovascular Diseases, Institute of Clinical Medicine, Faculty of Medicine, Vilnius University, Vilnius, Lithuania; 3 Ciencias de la Salud, Universidad Cardenal Herrera-CEU, CEU Universities, Moncada, Spain; 4 Department of Preventive Medicine and Public Health, School of Medicine, University of Valencia, Valencia, Spain; 5 Department of Internal Medicine, Consorci Hospital General Universitari de València, Valencia, Spain; 6 Research Unit of Biomedicine and Internal Medicine, Faculty of Medicine, University of Oulu, Oulu, Finland; 7 Biocenter Oulu, University of Oulu, Oulu, Finland; 8 Medical Research Center, Oulu University Hospital, Oulu, Finland; 9 Department of Pediatric Gastroenterology and Metabolic Diseases, Poznan University of Medical Sciences, Poznań, Poland; 10 Department of Internal Medicine, Hypertension and Vascular Risk Unit, Hospital de Sagunto, Valencia, Spain; 11 Foundation for the Promotion of Health and Biomedical Research in the Valencian Region (FISABIO), Valencia, Spain

**Keywords:** aortic stiffness, carotid-femoral pulse wave velocity, inflammation, uric acid, vascular aging

## Abstract

**Introduction:**

The importance of measuring vascular age has been emphasized in numerous studies, highlighting its critical role in precision medicine. This cross-sectional heterogeneity exploration study examined sex- and country-specific association of serum uric acid (SUA) and inflammation, measured as high sensitivity C-reactive protein (hs-CRP), and vascular aging across populations with diverse cardiovascular risks.

**Methods:**

This work analyzed data from three cohorts: 4,802 individuals from Sweden (SCAPIS – The Swedish CArdioPulmonary bioImage Study, *n* = 3,255), Lithuania (LitHiR – The Lithuanian High Cardiovascular Risk Prevention Program, *n* = 708), and Spain (The Sagunto Cohort, *n* = 838). Standard clinical and laboratory variables were used; aortic stiffness measured via carotid-femoral pulse wave velocity (cfPWV) (Sphygmocor), employing the foot-to-foot method in the right carotid and femoral arteries. In addition. Sex disaggregated analysis was performed.

**Results:**

The study involved individuals with a mean age of 56 (±8) years, 53% women, 48% never smokers, 41% with hypertension, and 20% with diabetes. In adjusted for cardiometabolic factors linear models, hs-CRP was associated with cfPWV only in Swedish men (*p* = 0.007), while SUA was associated with cfPWV in Swedish men (*p* = 0.001) and Lithuanian women (*p* = 0.029). In logistic regression, SUA predicted higher odds of cfPWV >10 m/s in Swedish men (OR 1.003, *p* = 0.041), and in Lithuanian women this prediction was of borderline significance (OR 1.004, *p* = 0.065). In Spanish women, the association with SUA was negative and of borderline significance (OR 0.995, *p* = 0.067). hs-CRP was not associated with cfPWV >10 m/s in adjusted models.

**Conclusion:**

This cross-sectional exploratory study provides evidence that SUA and hs-CRP are associated with vascular aging, although their predictive value should be interpreted in a sex- and country-specific context.

## 1 Introduction

The recognition of vascular age as a means of improving the precision of cardiovascular disease (CVD) risk assessment has been of long-standing focus of clinicians (1, 2). By providing an indirect measure of vascular remodeling, vascular age offers clinicians a valuable tool for identifying potential cardiovascular (CV) health risks before symptoms arise. The importance of measuring vascular age has been emphasized in numerous studies, highlighting its critical role in precision medicine (3).

Despite its potential utility, the adoption of vascular age in clinical practice has been slow (3). Many countries around the world rely on the SCORE2 – European High-Risk Chart, a well-established approach that has long guided practice in predicting CV risk (4), while there remains a growing need for more advanced approaches that incorporate vascular aging. Research on novel biomarkers, proteomics, and metabolomics as predictors of arterial stiffness is challenging to interpret, as the methods employed are currently too complex to be easily integrated into routine clinical practice (5, 6). Our work aims to shed light on simple, easily implementable biomarkers to estimate patient vascular age. We have selected serum uric acid (SUA) and high sensitivity C-reactive protein (hs-CRP) as potential candidates due to their significant interplay in CVD and their availability (7). The combination of these two biomarkers significantly improves CVD risk prediction. SUA is thought to contribute to CVD progression by triggering inflammatory responses, which in turn increase levels of IL-18 and hs-CRP (8) This complex interplay is further influenced by other key cardiometabolic risk factors, including age, obesity, hypertension and diabetes (9, 10).

The relationship between serum uric acid (SUA) and disease is complex, with both hypouricemia and hyperuricemia linked to adverse health outcomes. This “double-edged sword” phenomenon described by Wen et al. (11) explains the U-shaped association between SUA levels and morbidity, as well as cardiovascular and all-cause mortality (12, 13). Indeed, SUA’s protective properties are concentration dependent. While SUA levels within the normal range have protective effects, including blood pressure regulation, antioxidant properties, and anti-aging and neuroprotective benefits (11), hyperuricemia has pro-inflammatory properties that can lead to a thromboinflammatory state through activation of contact and complement systems, mainly by forming monosodium urate monohydrate (MSU) crystals (14) It furtherly leads to systemic inflammation (15) thrombosis (16), endothelial dysfunction (17), and vascular aging (18).

Both SUA and hs-CRP are widely used in clinical settings and have the potential to reveal important information on CV health. This cross-sectional exploratory study aims to investigate whether SUA levels, in combination with hs-CRP, perform similarly across multinational populations with varying CV risk in determining aortic stiffness, as measured by carotid-femoral pulse wave velocity. We hypothesize that SUA and hs-CRP are associated with aortic stiffness, and that their predictive value may be affected by sex and country.

## 2 Methods

### 2.1 Study participants

Three cohorts accounting for 4,802 individuals from different European Risk Regions based on SCORE2 and SCORE2-OP (4, 19) were analyzed. SCORE2 evolved to predict 10-year risk of first-onset CVD and incorporates sex- and country-specific approaches to CV risk estimation (4). SCORE2-OP provides calibrated risk prediction for elderly populations (19). Based on these models, European countries are categorized into four risk regions, where Spain is classified as a low risk region, Sweden as a moderate risk region, and Lithuania as a very high-risk region. The description of each study sample is presented below and summarized in Supplementary Table S1.

*SCAPIS* – *The Swedish CArdioPulmonary bioImage Study* (20) is a population-based study which randomly recruited Swedish residents in the 50–64 age group at six university hospitals. Nationwide, 30,154 individuals participated. 9,079 underwent measurement of arterial stiffness. For 3,518 of these individuals, examined at the Malmö site between January 2016 and March 2018, measurement of both SUA and hs-CRP was part of the screening program. Of those, 3,255 had complete information on lipid status, HbA1c, plasma glucose, plasma creatinine, anthropometric data, and blood pressure.

*The Sagunto Coho*rt was a prospective cohort study conducted in Sagunto, a city in the Valencia Region of Spain. The study aimed to investigate the relationship between CV risk factors and CVD. The Sagunto Cohort study included a sample of 1,500 participants aged 18–90 years, with suspected untreated primary hypertension at Sagunto Hospital (Sagunto, Spain), who were recruited between 2003 and 2006. Out of 900 individuals, 838 underwent arterial stiffness measurement and had serum uric acid and hs-CRP data.

*LitHiR – The Lithuanian High Cardiovascular Risk Prevention Program* (21) attendees who met inclusion criteria were included at primary care centers and examined at Vilnius University Hospital Santaros Klinikos (Vilnius, Lithuania). Age 40–54 years for men and 50–64 years for women, no previous history of CVD, but presence of metabolic syndrome according to the National Cholesterol Education Program Adult Treatment Panel III (NCEP ATP-III) guidelines were inclusion criteria for this study. Nationwide, 52,012 adults had been examined between 2006 and 2019. However, serum uric acid and hs-CRP were available for 709 individuals who were included in this analysis.

### 2.2 Measurements

Anthropometric measurements were taken in all three countries using a calibrated scale. Body weight (kg), waist circumference (WC, cm), and body height (cm) were measured. Body mass index (BMI) was calculated as weight divided by height square, kg/m^2^.

Different calibrated equipment was used for BP measurement. An automatic device (Omron M10-IT, Omron Health Care Co, Kyoto, Japan) was utilized in Sweden. BP was measured twice in each arm, and the mean value was used. In Spain validated, semiautomatic devices (OMRON M5-1, OMRON 705IT, Omron Health Care Co, Kyoto, Japan) were used and a manual sphygmomanometer was used in Lithuania. The mean BP value was calculated from three measurements on the left arm according to European Society of Hypertension (ESH) guidelines (22).

### 2.3 Arterial stiffness

The same methodological approach was used in all participants. Aortic stiffness was measured with Sphygmocor (v.7.01) (AtCor Medical Pty. Ltd. 1999–2002, Sydney, Australia) applanation tonometry, using the foot-to-foot method in the right carotid and femoral arteries for visualizing carotid-femoral pulse wave velocity (cfPWV). The simultaneous ECG registration was performed. The only difference was that in Sweden, the cuff-based device was used (23). Participants were advised to avoid caffeine, nicotine and alcohol intake several hours before the examination. The pulse travel distance was multiplied by 0.8.

### 2.4 Blood and urine samples

Fasting venous blood samples were collected. Serum uric acid (SUA), fasting plasma glucose (FPG), high-density lipoprotein cholesterol (HDL-c), low-density lipoprotein cholesterol (LDL-c), triglycerides (TG), serum creatinine, hs-CRP, glycated hemoglobin (HbA1c, available in Swedish and in Spanish participants), urinary albumin-to-creatinine ratio (UACR) (in Lithuanians and Spanish subjects) were analyzed.

Blood test results, including SUA measurements, obtained using the Abbott Architect ci8200 PLUS in Lithuania and the Roche Cobas 6000/8000 in Spain and Sweden, revealed comparable results, enabling reliable cross-platform analysis. Both analyzers meet standards for calibration with a certified reference material.

Kidney function was estimated by creatinine-based estimated glomerular filtration equations (eGFR) EKFC (The new creatinine-based European Kidney Function Consortium equation) (24). Conversion factors were applied to unify measurement values across countries.

### 2.5 Definitions

For this work, we utilized data gathered through questionnaires and clinical registries. Diabetes was defined as the use of antidiabetic drugs or previous known diagnosis or plasma glucose level above 7 mmoL/L and/or HbA1c level above 6.5%. Hypertension was defined as using antihypertensive medications or office systolic blood pressure (BP) ≥ 140 mmHg and/or diastolic BP ≥ 90 mmHg. Smoking was determined as “current smoker,” “former smoker” if smoking cessation happened 1 year before assessment, or “never” smoker.

Hyperuricemia is defined differently across countries, with varying SUA thresholds considered abnormal. In Sweden, SUA > 350 μmol/L in women 18–49 years old; >400 μmol/L in women 50 years or older; >480 μmol/L in adult men. In Spain, SUA ≥ 7.0 mg/dL (~416 μmol/L) for men and > = 6,5 mg/dL (~387 μmol/L) for women. In Lithuania, SUA values >357 μmol/L in women and >428 μmol/L in men.

Swedish participants represented the largest subgroup and exhibited the lowest SUA levels; therefore, their sex-specific SUA quartiles were used as a reference value for grouping Lithuanian and Spanish patients. 1st quartile (SUAq1) values < 225 μmol/L (women) or < 301 μmol/L (men); 2nd quartile (SUAq2) – 225-265 μmol/L (women) or 301–342 μmol/L (men); 3rd quartile (SUAq3) – 265-309 μmol/L (women) or 342–387 μmol/L (men); 4th quartile (SUAq4) – values >309 μmol/L (women) or >387 μmol/L (men). The distribution of sex-specific SUA quartiles is presented in Supplementary Figure 1.

Elevated UACR excretion was determined as ≥3 mg/mmoL in Lithuanians and Spaniards.

### 2.6 Outcome and exposure variables

cfPWV was considered as an outcome variable. In linear models, crude values were used. In logistic regression, we utilized cfPWV values above 10 m/s, as per the guidelines of the ESH (22).

SUA and hs-CRP were exposure variables.

### 2.7 Statistical analysis

Normally distributed variables are presented as means and standard deviations, non-parametric – as medians and interquartile ranges and categorical – as percentages and case numbers. The hs-CRP values did not follow a normal distribution; therefore, a natural logarithmic transformation (ln) of hs-CRP was applied, with 1-unit increase in ln(hs-CRP) corresponding approximately to a 2.72-fold increase in hs-CRP. Additionally, due to the high variability in SUA, the values were rescaled, and SUA was analyzed per 50 μmol/L increase (SUA_50_). The transformed data were analyzed using both linear and logistic regression models. Analyses were stratified by sex. cfPWV z-scores (cfPWVz) were calculated to plot cross-country comparisons of aortic stiffness, using recently published reference values derived from white male and female participants in the longitudinal Austrian Lung, hEart, sociAl, boDy (LEAD) study (25). cfPWVz use the average cfPWV of healthy individuals of the same age and sex as a comparator. The equation for cfPWVz:


$$\text{cfPWVz}=\frac{\text{cfPWV}(\text{measured})-\text{cfPWV}(\text{expected})}{\mathrm{SD}(\text{expected})}$$

Where cfPWV(measured) is a raw value, cfPWV(expected) is the reference value, and SD (expected) is the population standard deviation published by Azizzadeh et al. (25).

Sex disaggregated linear regression analysis defined the association between cfPWV (outcome), hs-CRP (exposure) and SUA (exposure). Interaction analyses were performed to assess potential interactions between SUA and cohort, hs-CRP and cohort, and the three-way interaction among SUA, hs-CRP, and cohort. For this purpose, centralized values of SUA and hs-CRP were calculated by subtracting the overall mean value from each data point (SUA_C and hs-CRP_C). Variables SUA_C, hs-CRP_C, SUA_C*cohort, hs-CRP_C*cohort, and SUA_C*hs-CRP_C*cohort were then entered into a linear regression model with cfPWV as the outcome. Both SUA and hs-CRP showed significant interactions with cohort (SUA_C*cohort: *β* = 0.002, SE = 0.0004, *p* < 0.001; hs-CRP_C*cohort: *β* = 0.134, SE = 0.028, *p* < 0.001; SUA_C*hs-CRP_C*cohort: *β* = 0.0004, SE = 0.0001, *p* < 0.001). Therefore, data were not pooled, and each country was analyzed separately. No collinearity between SUA and hs-CRP was detected, as the Variance Inflation Factor (VIF) in the unadjusted model was 1.08 for women and 1.02 for men. VIF’s were further employed in nested linear models (Models 1 to 3), incorporating the covariates listed below. This approach helped identify factors mediating or mitigating the relationship between outcome and exposure.

Model 1: Exposure variables adjusted for age and BMI.

Model 2: Model 1 adjusted for eGFR, heart rate (HR), pulse pressure (PP).

Model 3: Model 2 adjusted for diabetes, smoking, FPG, TG, antihypertensive and lipid-lowering treatment.

A sensitivity analysis was performed using cfPWVz as the outcome to assess the robustness of the results. These results are presented in Supplemental materials.

Sex specific bivariate logistic regression with an outcome variable included similar nested Models 1 to 3 as in linear regression. The outcome and exposure variables as defined previously in the paper.

SPSS 29.0 software was used for statistical analysis. A *p*-value below 0.05 was considered statistically significant.

## 3 Results

**Figure 1:**
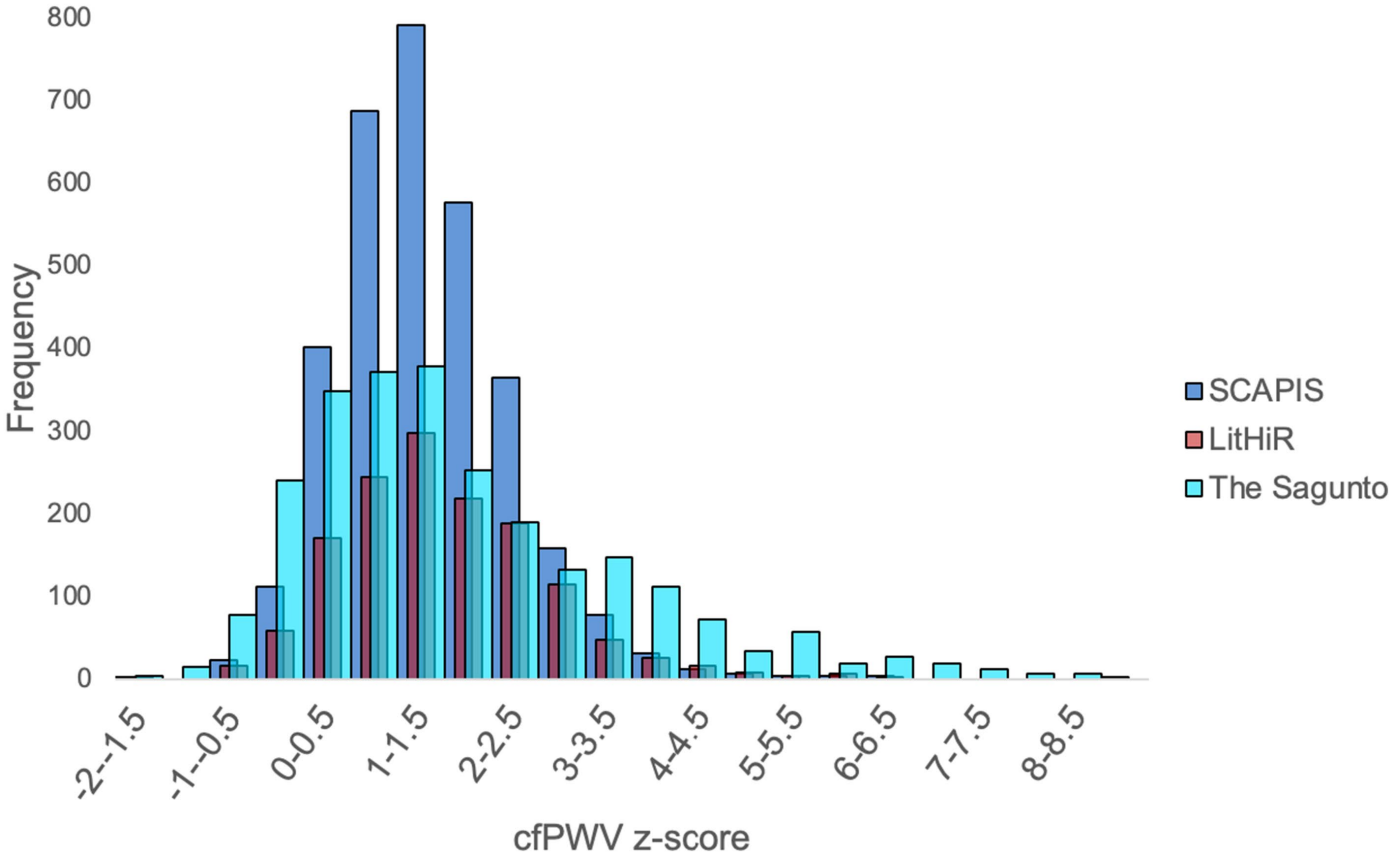
Country based histogram for cfPWV *z*-score. The *y*-axis corresponds cfPWVz in increments of 0.5; the *x*-axis indicates the frequency.

Table 1 summarizes the profile of study participants. The Lithuanian cohort had the highest proportion of women. Despite their older age, Swedish participants had better cardiometabolic health. They had the lowest prevalence of hypertension, diabetes, were less overweight, were less frequently treated with lipid-lowering drugs, had the lowest SUA and hs-CRP, and had the lowest hemodynamic parameters. Indeed, the histogram of cfPWV was shifted to the right (Figure 1), with largely overlapping country-based cfPWVz values between 0 and +2SD. Spaniards, however, had the highest prevalence of cfPWVz values above +2SD (*p* < 0.001).

**Table 1:** Description of variables.

Variables	SCAPIS			LitHiR			The Sagunto		
	*N* = 3,255			*N* = 709			*N* = 838		
	WOMEN (*N* = 1,682)	MEN (*N* = 1,573)	*p*-value	WOMEN (*N* = 444)	MEN (*N* = 265)	*p*-value	WOMEN (*N* = 424)	MEN (*N* = 414)	*p*-value
	% (*n*)	% (*n*)		% (*n*)	% (*n*)		% (*n*)	% (*n*)	
Smoking			0.408			<0.001			<0.001
Never	44.8 (739)	44.8 (681)		82.9 (368)	59.8 (158)		41.6 (123)	24.6 (83)	
Former	16.1 (266)	17.8 (270)		15.1 (67)	32.2 (85)		37.8 (112)	33.1 (112)	
Current	39.0 (643)	37.4 (568)		2.0 (9)	8.0 (21)		20.6 (61)	42.3 (143)	
Hypertension			0.327			0.003			<0.001
No	76.9 (1243)	75.4 (1126)		10.6 (47)	18.5 (49)		32.3 (136)	15.7 (64)	
Yes	23.1 (373)	24.6 (367)		89.4 (397)	81.5 (216)		67.7 (285)	84.3 (344)	
Diabetes			<0.001			0.031			0.23
No	93.5 (1573)	88.9 (1397)		77.4 (343)	84.2 (223)		89.6 (380)	87.0 (360)	
Yes	6.5 (109)	11.1 (175)		22.6 (100)	15.8 (42)		10.4 (44)	13.0 (54)	
Previous stroke			0.092			—			0.138
No	98.5 (1657)	97.7 (1537)		100 (444)	100 (265)		97.9 (415)	96.1 (398)	
Yes	1.5 (25)	2.3 (36)		0 (0)	0 (0)		2.1 (9)	3.9 (16)	
CHD			0.261			0.883			0.265
No	99.2 (1603)	98.8 (1475)		99.5 (441)	99.6 (264)		97.9 (415)	96.1 (400)	
Yes	0.8 (13)	1.2 (18)		0.5 (2)	0.4 (1)		2.1 (9)	3.9 (14)	
Anti-hypertensive therapy			0.239			<0.001			0.251
No	80.7 (1358)	79.1 (1244)		25.9 (115)	47.9 (127)		75.5 (320)	72.0 (298)	
Yes	19.3 (324)	20.9 (329)		74.1 (329)	52.1 (138)		24.5 (104)	28.0 (116)	
Lipid-lowering therapy			0.004			0.448			0.021
No	93.9 (1572)	90.7 (1427)		75.2 (334)	77.7 (206)		79.5 (337)	72.7 (301)	
Yes	6.1 (110)	9.3 (146)		24.8 (110)	22.3 (59)		20.5 (87)	27.3 (113)	

Variable	Mean ± SD	Mean ± SD	*p*-value	Mean ± SD	Mean ± SD	*p*-value	Mean ± SD	Mean ± SD	*p*-value
Age (years)	57.4 ± 4.2	57.5 ± 4.3	0.460	57.5 ± 4.2	47.1 ± 4.2	<0.001	52.0 ± 14.6	51.3 ± 14.0	0.512
Height (m)	1.65 ± 0.06	1.79 ± 7.2	<0.001	1.64 ± 0.06	1.80 ± 0.06	<0.001	1.58 ± 7.1	1.71 ± 7.1	<0.001
Weight (kg)	72.5 ± 12.9	88.9 ± 13.7	<0.001	86.1 ± 14.2	101.0 ± 12.8	<0.001	71.9 ± 15.4	86.5 ± 14.7	<0.001
BMI (kg/m^2^)	26.5 ± 4.6	27.7 ± 3.8	<0.001	31.9 ± 4.9	31.2 ± 3.7	0.05	28.9 ± 5.6	29.4 ± 4.2	0.148
Waist circumference (cm)	89.2 ± 11.6	100.9 ± 10.8	<0.001	100.5 ± 10.5	106.3 ± 8.7	<0.001	88.8 ± 15.5	99.2 ± 14.2	<0.001
Serum uric acid (μmol/L)	270.0 ± 63.8	346.9 ± 68.2	<0.001	333.4 ± 81.4	400.2 ± 81.4	<0.001	280.3 ± 77.6	374.8 ± 81.9	<0.001
ln-hsCRP	0.31 ± 0.87	0.27 ± 0.86	0.247	0.58 ± 0.97	0.49 ± 0.93	0.272	0.69 ± 1.10	0.61 ± 1.04	0.278
Glycemia (mmol/L)	5.46 ± 1.07	5.76 ± 1.51	<0.001	6.54 ± 1.79	6.43 ± 1.77	0.443	5.57 ± 1.47	5.77 ± 1.26	0.034
HDL-C (mmol/L)	1.88 ± 0.53	1.44 ± 0.43	<0.001	1.36 ± 0.31	1.08 ± 0.24	<0.001	1.57 ± 0.38	1.25 ± 0.32	<0.001
LDL-C (mmol/L)	3.60 ± 0.93	3.59 ± 1.00	0.782	3.88 ± 1.16	3.70 ± 1.15	0.038	3.14 ± 0.92	3.07 ± 0.92	0.236
Triglycerides (mmol/L)	1.16 ± 0.79	1.49 ± 1.01	<0.001	1.93 ± 1.36	2.81 ± 4.41	<0.001	1.26 ± 0.65	1.54 ± 0.97	<0.001
HbA1c (mmol/mol)	36.9 ± 5.6	37.7 ± 8.6	0.003	No data			28.9 ± 19.5	29.6 ± 18.8	0.588
Creatinine (μmol/L)	69.2 ± 10.4	86.7 ± 12.9	<0.001	66.5 ± 8.5	81.0 ± 12.1	<0.001	66.6 ± 17.9	87.5 ± 19.6	<0.001
eGFR (EKFC, mL/min/1.73m^2^)	79.9 ± 11.2	82.0 ± 10.7	<0.001	83.0 ± 10.1	96.2 ± 10.9	<0.001	87.9 ± 19.7	86.1 ± 16.8	0.165
Systolic BP (mmHg)	123.0 ± 15.5	127.7 ± 13.9	<0.001	134.3 ± 15.7	131.5 ± 12.1	0.014	137.9 ± 17.7	139.6 ± 16.7	0.146
Diastolic BP (mmHg)	73.1 ± 8.8	77.2 ± 8.9	<0.001	80.7 ± 8.4	84.5 ± 8.0	<0.001	79.2 ± 10.6	81.9 ± 12.2	<0.001
Pulse pressure (mmHg)	49.9 ± 10.1	50.5 ± 8.9	0.072	53.5 ± 12.1	47.0 ± 8.6	<0.001	58.7 ± 14.5	57.7 ± 13.2	0.307
cSBP (mmHg)	113.7 ± 14.1	116.8 ± 12.7	<0.001	125.8 ± 14.2	119.5 ± 11.7	<0.001	133.2 ± 20.6	130.9 ± 18.8	0.095
cDBP (mmHg)	74.0 ± 8.9	78.1 ± 9.1	<0.001	81.5 ± 9.2	85.3 ± 8.1	<0.001	84.2 ± 10.7	84.0 ± 11.1	0.782
HR (bpm)	61.9 ± 8.7	60.7 ± 9.6	<0.001	65.9 ± 9.9	64.6 ± 9.0	0.082	77.4 ± 13.3	71.3 ± 12.7	<0.001
cfPWV (m/s)	8.0 ± 1.2	8.6 ± 1.2	<0.001	8.76 ± 1.49	8.06 ± 1.29	<0.001	8.7 ± 2.4	8.7 ± 2.2	0.952
cfPWV z-score	1.10 ± 0.86	1.56 ± 0.91	<0.001	1.67 ± 1.10	1.16 ± 0.95	<0.001	1.65 ± 1.77	1.64 ± 1.65	0.952

	Median (IQR)	Median (IQR)	*p*-value	Median (IQR)	Median (IQR)	*p*-value	Median (IQR)	Median (IQR)	*p*-value
UACR	No data			0.58 (0.42)	0.50 (0.36)	<0.001	5.00 (10.0)	4.00 (7.0)	0.001

Normally distributed variables are presented as mean value (SD), non-parametric as medians (IQR) and categorical as percentage % (case numbers).

CHD, coronary heart disease, SUA, serum uric acid; hs-CRP, high sensitivity C-reactive protein; FPG, fasting plasma glucose; HDL-c; high density lipoprotein; LDL-c, low density lipoprotein; TG, triglycerides; HbA1c, glycolyzed hemoglobin; EKFC, The new creatinine-based European Kidney Function Consortium equation; UACR, urine albumin-to-creatinine ratio; SBP, systolic blood pressure, (p) peripheral, (c) central; DBP, diastolic blood pressure, (p) peripheral, (c) central; PP, pulse pressure; cfPWV, carotid-femoral pulse wave velocity; cfPWVz, z-score of cfPWV; HR, heart rate.

Prevalence of hyperuricemia defined according to local reference values in Sweden, Lithuania and Spain was inconsistent (Supplementary Table S2). A trend toward an increased prevalence of hyperuricemia in Lithuanian women and Spanish men was observed based on country-specific guidelines. Interestingly, irrespective of definition, individuals classified as having hyperuricemia had significantly higher hs-CRP, cfPWV, cSBP, central diastolic BP (cDBP) and HR but lower creatinine-based eGFR (according to all equations used, not shown in a table) (*p* < 0.001).

### 3.1 Comparison of serum uric acid quartiles

ANOVA revealed a “J”-shaped association, with the highest cfPWV in the highest SUA quartile (Q4) among Swedish, Lithuanian, and Spanish women (*p* < 0.001) (Supplementary Figure 1). In men, no differences in cfPWV across SUA quartiles were observed, except for a near “U”-shaped pattern in Spaniards (*p* < 0.001). hs-CRP distribution was more complex, with increasing values across SUA quartiles in Swedish, Lithuanian, and Spanish women, but with a less clear trend in men. Younger Spanish (*p* = 0.026) and Lithuanian men (*p* < 0.001), as well as older Spanish and Swedish women, were more frequently in the highest SUA quartile (*p* < 0.001 for both groups). Age did not differ across SUA quartiles in Lithuanian women (*p* = 0.840) and Swedish men (*p* = 0.669). BMI increased stepwise (*p* < 0.001), and pulse pressure showed a right-peaked distribution (all *p* < 0.001) (Supplementary Figure 1). Kidney function, determined by creatinine-based eGFR, was lower in the highest SUA quartile across countries and sexes (all *p* < 0.001; in Spanish men *p* = 0.002), except in Lithuanian men (*p* = 0.315).

### 3.2 Inflammation and uric acid in prediction of aortic stiffness

**Figure 2:**
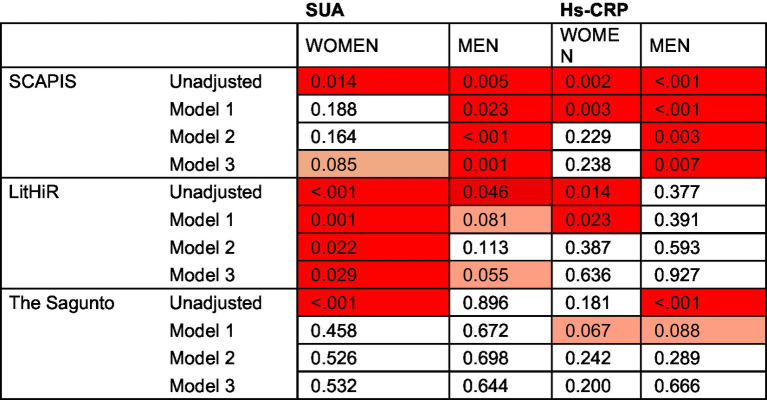
Heatmaps displaying *p*-values from linear regression models predicting cfPWV. *p*-values <0.05 are shown in red, 0.05–0.10 in light red, and ≥0.10 in white.

Sex-stratified linear regression analyses showed country-specific associations between cfPWV, hs-CRP, and SUA (Table 2). In unadjusted models, cfPWV was significantly associated with both hs-CRP and SUA in Swedish women and men (*p* = 0.014 and *p* = 0.005, respectively), and in Lithuanian women and men (*p* < 0.001 and *p* = 0.046, respectively). In contrast, cfPWV was associated only with SUA in Spanish women (*p* < 0.001), and with hs-CRP in Spanish men (*p* < 0.001). In Model 1, associations were attenuated: a ~ 2.7 fold increase in hs-CRP was associated with 0.17 m/s higher cfPWV in Lithuanian women and a 0.11 m/s higher cfPWV in Swedish women (*p* = 0.003 and *p* = 0.023, respectively), as well as a 0.14 m/s higher cfPWV in Swedish men (*p* < 0.001). Meanwhile, each 50 μmol/L increase in SUA remained significantly associated with a 0.14 m/s higher cfPWV in Lithuanian women (*p* = 0.001) and a 0.05 m/s higher cfPWV in Swedish men (*p* = 0.023). In Models 2 and 3, a ~ 2.7 fold increase in hs-CRP was associated with a 0.09–0.10 m/s higher cfPWV only in Swedish men (*p* = 0.007, Model 3), while each 50 μmol/L increase in SUA was associated with 0.07 m/s higher cfPWV in Swedish men (*p* = 0.001, Model 3) and a 0.09 m/s higher cfPWV in Lithuanian women (*p* = 0.029, Model 3). Complete information on regression coefficients is shown in Supplementary Table S3. The heat maps of *p*-values are presented in Figure 2.

**Table 2:** Association between cfPWV, serum uric acid and C-reactive protein.

Origin	Variable	Women				Men			
		B	SE	*p*-value	VIF	B	SE	*p*-value	VIF
Unadjusted									
SCAPIS	SUA_50_	0.058	0.023	0.014	1.122	0.064	0.023	0.005	1.04
	hs-CRP	0.105	0.034	0.002	1.122	0.174	0.036	<0.001	1.04
LitHiR	SUA_50_	0.160	0.044	<0.001	1.109	0.099	0.050	0.046	1.05
	hs-CRP	0.184	0.074	0.014	1.109	0.077	0.086	0.377	1.05
The Sagunto	SUA_50_	0.464	0.073	<0.001	1.062	0.009	0.066	0.896	1.02
	hs-CRP	0.137	0.103	0.181	1.062	0.533	0.104	<0.001	1.02
Model 1									
SCAPIS	SUA_50_	0.038	0.024	0.188	1.225	0.052	0.023	0.023	1.12
	hs-CRP	0.108	0.036	0.003	1.343	0.140	0.036	<0.001	1.12
LitHiR	SUA_50_	0.139	0.043	0.001	1.167	0.090	0.052	0.081	1.18
	hs-CRP	0.172	0.075	0.023	1.269	0.075	0.087	0.391	1.10
The Sagunto	SUA_50_	0.043	0.058	0.458	1.286	−0.022	0.051	0.672	1.09
	hs-CRP	0.149	0.081	0.067	1.271	0.142	0.083	0.088	1.15
Model 2									
SCAPIS	SUA_50_	0.029	0.021	0.164	1.315	0.078	0.021	<0.001	1.25
	hs-CRP	0.038	0.032	0.229	1.374	0.097	0.032	0.003	1.13
LitHiR	SUA_50_	0.095	0.041	0.022	1.318	0.085	0.053	0.113	1.28
	hs-CRP	0.061	0.071	0.387	1.324	0.046	0.086	0.593	1.12
The Sagunto	SUA_50_	0.039	0.062	0.526	1.573	−0.019	0.050	0.698	1.18
	hs-CRP	0.094	0.080	0.242	1.350	0.083	0.078	0.289	1.16
Model 3									
SCAPIS	SUA_50_	0.037	0.021	0.085	1.030	0.070	0.022	0.001	1.28
	hs-CRP	0.038	0.032	0.238	1.070	0.089	0.062	0.007	1.15
LitHiR	SUA_50_	0.092	0.042	0.029	1.032	0.102	0.053	0.055	1.36
	hs-CRP	0.034	0.072	0.636	1.050	−0.008	−0.006	0.927	1.16
The Sagunto	SUA_50_	0.041	0.065	0.532	1.021	−0.026	−0.056	0.644	1.30
	hs-CRP	0.115	0.090	0.200	1.044	0.038	0.018	0.666	1.19

Sex stratified linear regression.

cfPWV dependent variable. Complete information on regression coefficients for all variables is provided in Supplementary Table S3. SUA_50_ corresponds SUA values per 50 μmol/L increase.

hs-CRP was analyzed on the logarithmic scale. A 1-unit increase in ln(hs-CRP) ≈ 2.72-fold increase in hs-CRP.

Model 1: SUA and Ln(hs-CRP) adjusted for age and BMI.

Model 2: + adjusted for eGFR, heart rate, PP.

Model 3: + adjusted for diabetes, smoking, FPG, TG, antihypertensive and lipid-lowering treatment.

Sensitivity analyses with cfPWVz as the outcome yielded identical *p*-values, although the associations were weaker (Supplementary Table S4).

**Figure 3:**
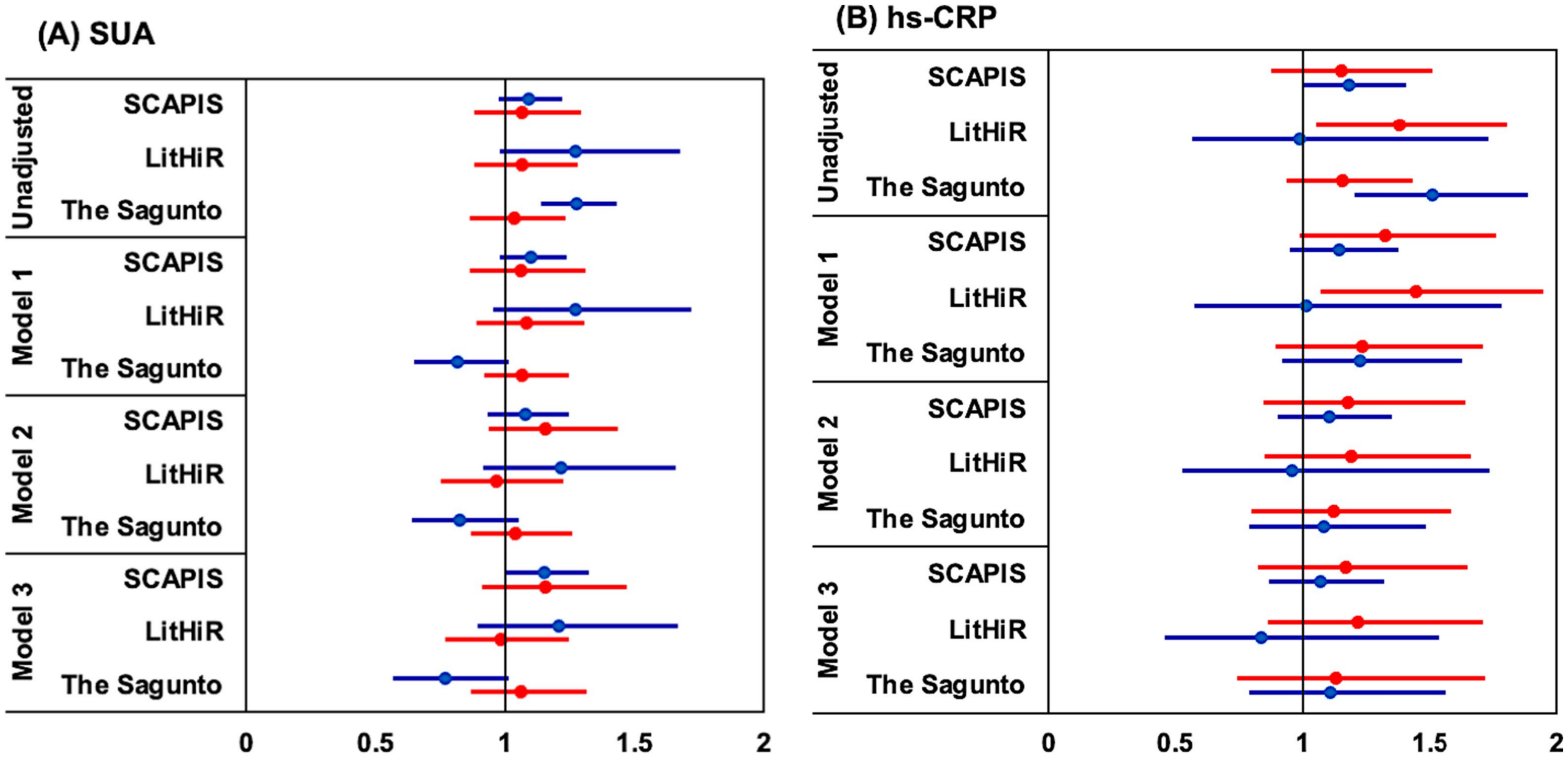
Logistic regression analysis for predicting increased cfPWV. Forrest plots for odds ratios of serum uric acid and high-sensitivity CRP. Odds in women depicted in “red,” in men – in “blue.” **(A)** SUA, presented per 50 μmol/L increase **(B)** hs-CRP, logarithmic scale, a 1-unit increase corresponds to ≈ 2.72-fold increase in hs-CRP.

In unadjusted logistic regression, each 50 μmol/L increase in SUA and 2.7 fold increase in hs-CRP predicted higher odds (OR) of cfPWV >10 m/s in Lithuanian women (OR 1.272, *p* = 0.002 for SUA; OR 1.378, *p* = 0.021 for hs-CRP, Figures 3A,B). Associations were also observed with higher hs-CRP levels in Spanish men (OR 1.506, *p* = 0.001) and were borderline significant in Swedish men (OR 1.182, *p* = 0.057), see Figure 3A. In Spanish women, only 50 μmol/L increase in SUA could predict 27% increase in cfPWV (OR 1.274, *p* = 0.001), see Figure 3B. After adjustment for age and BMI, hs-CRP remained significant in Lithuanian women (OR 1.443, *p* = 0.016, Figure 3B). Interestingly, each 50 μmol/L decrease in SUA was associated with 19% higher cfPWV in Spanish women (OR 0.815, *p* = 0.043), whereas each 50 μmol/L increase in SUA predicted 27% higher cfPWV in Lithuanian women (OR 1.270, *p* = 0.004) (Model 1; Table 2; Figures 3A,B). With further adjustments (Models 2–3; Table 2; Figure 3A), SUA predicted cfPWV >10 m/s in Swedish men (Model 2: OR 1.156, *p* = 0.034; Model 3: OR 1.155, *p* = 0.041) and in Lithuanian women (Model 2: OR 1.216, *p* = 0.047; Model 3: OR 1.205, *p* = 0.065). In Spanish women, the association with SUA remained negative and of borderline significance (OR 0.766, *p* = 0.067). hs-CRP was not associated with cfPWV >10 m/s in adjusted models.

In Model 3, additional independent predictors of cfPWV >10 m/s included age, BMI, and heart rate in Swedes and Spaniards, and pulse pressure in Swedes. Sex-specific associations were observed for higher glucose levels and lipid-lowering therapy in Swedish women; eGFR in Swedish men; heart rate, pulse pressure, and smoking in Lithuanian women; and higher glucose in Spanish men. No significant predictors were found in Lithuanian men. Detailed coefficients are provided in Supplementary Table S5.

## 4 Discussion

This cross-sectional exploratory study demonstrates that two biomarkers—serum uric acid and high-sensitivity C-reactive protein— serve as indicators of vascular aging. Strength and direction of this relationship, however, varies by sex, country and patient selection. Linear regression revealed a more nuanced profile, with elevated levels of SUA and hs-CRP showing stronger associations with higher cfPWV compared with logistic regression using a cfPWV cutoff of >10 m/s across the cohorts. Notably, the most consistent trends across models were observed in Swedish men and Lithuanian women for the association between SUA and cfPWV, and in Swedish men alone for the association between hs-CRP and cfPWV. Interestingly, contrasting patterns emerged between countries: in Spanish women, lower SUA levels were linked to cfPWV >10 m/s, whereas in Lithuanian women, higher SUA levels were associated with cfPWV >10 m/s.

Evidence indicates that PWV varies across countries. These differences are likely influenced by variations in the prevalence of vascular risk factors, many of which are related to PWV, as well as dietary habits, physical activity, heart rate, and racial, ethnic, and genetic backgrounds. Supporting a genetic contribution, a substantial heritability of cfPWV of approximately h^2^ ≈ 0.4 was reported in 1,480 participants from the Framingham Offspring Cohort, with a mean age of approximately 60 years (9).

In the era of personalized medicine, SUA and hs-CRP should be interpreted in an individual context. Although Spain has consistently demonstrated a lower burden of cardiovascular disease—as reflected in the Global Burden of Cardiovascular Disease and Risk report from 1990 to 2021—this study recruited subjects with suspected untreated primary hypertension. These subjects more frequently exhibited cfPWV z-scores above +2 SD, indicating markedly increased arterial stiffness due to early vascular aging, despite Spain having one of the lowest age-standardized disability-adjusted life years (DALYs) per 100,000 individuals (range: 1305.1–1871.8). By contrast, Sweden and Lithuania demonstrate significantly higher DALY rates, with ranges of 1871.9–2907.5 and 4750.5–5465.8, respectively (26).

Spanish women with lower SUA levels showed a trend toward increased odds of having cfPWV above 10 m/s. In contrast, Lithuanian women demonstrated the opposite relationship. A recent report by Huang Y et al. (27) demonstrated that in hypertensive individuals both low and high SUA levels are associated with all-cause mortality. These findings are supported by previous studies, which specifically in women demonstrated a U-shaped relationship between SUA levels and 10-year CVD risk (27, 28). Indeed, Spanish women were selected from a cohort with suspected hypertension and had elevated hs-CRP levels, indicating underlying low-grade inflammation, compared with the Swedish and Lithuanian women. This pro-inflammatory state promotes oxidative stress, i.e., increased production of reactive oxygen species (ROS). Under such conditions, uric acid is consumed to fulfill its antioxidative role (29). Other possibility involves factors not captured in this study, such as increased urate excretion due to glucosuria, well described in diabetes (30), or the use of SUA-lowering medications. The lack of similar findings in Spanish and Lithuanian women may be due to selection bias. Lithuanian women already had established metabolic syndrome, in which the protective properties of uric acid may have been depleted, and the higher SUA is associated with stiffer arteries. Swedish women were drawn from the general population and had a very low burden of hypertension and obesity.

In both Spanish and Lithuanian men, SUA showed limited accuracy in predicting cfPWV above 10 m/s. This may be due to the influence of other cardiometabolic risk factors—such as sedentary lifestyle, purine-rich diet, and alcohol consumption—which may have outweighed the contribution of SUA in the Sagunto and LitHiR cohorts. In Spanish men, cfPWV >10 m/s was more strongly predicted by age, BMI, pulse pressure, and fasting plasma glucose. By contrast, none of these factors were significant in Lithuanian men, many of whom entered the study with low health literacy and already advanced vascular aging (31). The observed relationship between higher SUA and aortic stiffness in Swedish men may be a reflection of statistical power (i.e., a larger sample improves the accuracy of the association) and a lower likelihood of confounding from uric-acid–lowering medications.

The difference across cohorts may also reflect ongoing debate about the optimal cfPWV cut-off for defining cardiovascular risk, since current ESH guidelines do not account for sex or ethnicity (20). A multicenter study spanning Greece, Italy, Portugal, Belgium, Lithuania, and the USA reported substantial variation in mean cfPWV values between countries (32). In our analysis, Spanish participants and Lithuanian men had the highest cfPWV z-scores, but the effect of inflammation on cfPWV was particularly evident in the Swedish men (linear models). These findings suggest that country- and sex-specific thresholds may better capture population differences. For example, the inflammatory signal observed in Swedish men would be overlooked if the universal 10 m/s cut-off were applied.

In Swedish men, a stronger linear association between hs-CRP and cfPWV was seen despite overall lower hs-CRP levels compared with other countries. This may be explained by their older age, allowing for a greater cumulative impact of inflammation on arterial remodeling. SUA in this group appeared to contribute both directly and indirectly through its influence on hs-CRP (7). In contrast, Lithuanian and Spanish men and women had higher hs-CRP levels at baseline, suggesting a more pronounced inflammatory background that may have diluted its association with arterial stiffness.

In subsamples of Lithuanian men and Spanish men and women, where inflammation was less predictive, traditional risk factors—age, BMI, heart rate, pulse pressure and glycemia—were independently associated with cfPWV. This points to population-specific hallmarks of vascular aging and suggests that established cardiometabolic factors can outweigh the predictive value of SUA and hs-CRP.

Overall, these findings underscore the complexity and individualization in determining vascular aging, which cannot be explained by a single marker. Our results echo previous evidence that both low BMI (observed in Swedish men and women) and high BMI (observed in Spanish men and women) may accelerate vascular aging. As shown in earlier studies, low BMI does not always equate to metabolic health, and both obesity and underweight in older adults have been linked to shorter life expectancy due to accelerated biological aging (33).

### 4.1 Limitations and strengths

This study has several limitations that should be acknowledged. First, its cross-sectional exploratory design, in which heterogeneous cohorts with different inclusion criteria were compared, may introduce selection, measurement, confounding, temporal, and social desirability biases. The Swedish cohort represented a random sample from the general population, whereas the Lithuanian and Spanish cohorts comprised individuals already identified as being at higher cardiovascular risk. Inter-operator variability in central hemodynamic assessment might also contributed to measurement bias. In addition, differences in equipment and measurement units required the use of conversion factors for dataset harmonization, which may have introduced minor inaccuracies. Social desirability bias may have distorted the reported prevalence of smoking across the cohorts. Finally, information on dietary habits, alcohol consumption, and the use of uric acid–lowering medication was not available.

A key strength of our study is that we analyzed a comprehensive dataset encompassing individuals with diverse demographic characteristics and varying cardiovascular risk profiles. Vascular aging was assessed using cfPWV, the gold standard measurement of arterial stiffness. Finally, the study’s findings have clear translational relevance to clinical practice, as the biomarkers investigated are inexpensive, widely available, and easily implemented in routine care.

### 4.2 Clinical implications

The clinical implications of this cross-sectional study should be interpreted with caution, as longitudinal outcome data are not available. Although many previous works showed that anti-inflammatory and uric acid–lowering therapies in individuals with hypertension have been associated with improved CV outcomes, the present findings might suggest that this approach may not be uniformly beneficial across all populations. For example, in Spanish women, lower SUA levels were associated with increased cfPWV, raising the possibility that SUA reduction may not always be advantageous. Similarly, it remains uncertain whether intervening in otherwise healthy individuals, such as Swedish men, based on cfPWV levels alone, prior to the development of gout, kidney disease, or markedly elevated SUA, would lead to meaningful CV benefit. Future studies in large, population-based cohorts are needed to replicate these findings and to better identify subgroups with low to moderate cardiovascular risk in whom very high or very low SUA levels, in conjunction with inflammation, may warrant targeted intervention.

## 5 Conclusion

This cross-sectional exploratory study provides evidence that SUA and hs-CRP are associated with vascular aging, although their predictive value should be interpreted in a sex- and country-specific context. Both elevated and reduced SUA levels were associated with increased aortic stiffness in men from a moderate cardiovascular risk setting (Sweden) and in women from populations with either very high risk (Lithuania) or suspected untreated hypertension (Spain). hs-CRP emerged as a particularly sensitive biomarker in Swedish men, where lower-grade inflammation was present, especially when analyzed in linear models without reliance on fixed cut-off values. Importantly, both SUA and hs-CRP must be considered alongside established cardiometabolic risk factors—including age, sex, body mass index, fasting glucose, heart rate, and pulse pressure—to fully capture their role in vascular aging.

## Data Availability

The raw data supporting the conclusions of this article will be made available by the authors, without undue reservation, under reasonable request.
