# Supplementary material for: Sex- and country-specific associations of hyperuricemia and inflammation with vascular aging across populations with diverse cardiovascular risks
Source: Front Med (Lausanne). 2026 Jan 21;12:1737935. doi: 10.3389/fmed.2025.1737935 (PMC12867889; doi:10.3389/fmed.2025.1737935)
Supplement: Supplementary file 1 [file Table_1.DOCX]

Supplementary Material

**Supplemental Table S1** Cohort description

| **Study Name** | **Country** | **Recruitment Period** | **Age Range (years)** | **Participants (Total)** | **cfPWV Measured** | **SUAa nd hs-CRP Measured** | **Exclusion Criteria** |
| --- | --- | --- | --- | --- | --- | --- | --- |
| **SCAPIS**  Swedish CArdioPulmonary bioImage Study | Sweden | 2016 – 2018 (Malmö site) | 50–64 | 30,154 | 9,079 | 3,255  (Malmö site) | Inability to understand Swedish, severe cognitive impairment, severe chronic disease |
| **LitHiR**  Lithuanian High Cardiovascular Risk Prevention Program | Lithuania | 2006 – 2019 | Men: 40–54, Women: 50–64 | 52,012 | Not specified | 709 | Clinically evident CVD, advanced cancer, other advanced somatic disease |
| **Sagunto Cohort** | Spain | 2003 – 2006 | 40–75 | 1,500 | 838 | 838 | History of CVD, cancer, secondary hypertension, liver/kidney failure, advanced heart failure (NYHA III–IV), infection, gout, renal stones, uric acid medication use |

**Supplemental Table S2** Prevalence of hyperuricemia according to country-specific guidelines

|  |  |  | Sweden† | Lithuanian† | Spain† |
| --- | --- | --- | --- | --- | --- |
| Prevalence of hyperuricemia (%, n) | Swedish | Women | 2.9 (49) | 8.6 (144) | 4.4 (74) |
|  |  | Men | 3.9 (62) | 0.8 (170) | 13.9 (218) |
|  | Lithuanian | Women | 17.8 (79) | 34.5 (153) | 21.6 (96) |
|  |  | Men | 16.2 (43) | 32.1 (85) | 38.1 (101) |
|  | Spanish | Women | 9.9 (42) | 14.2 (60) | 9.7 (41) |
|  |  | Men | 9.7 (40) | 23.4 (97) | - 1. (115) |

† The definition of hyperuricemia in Sweden corresponds SUA >350 µmol/L in women (18-49 years); >400 µmol/L in women (>=50 years old); >480 µmol/L in adult men; in Spain, SUA values >= 7.0 mg/dL (~416 µmol/L) for men and >=6,5 mg/dL (~387 µmol/L) for women; in Lithuania, SUA values >357 µmol/L in women and >428 µmol/L in men.

**Supplemental Table S3** Complete information on linear regression models with cfPWV as the outcome

|  | | WOMEN | | | | MEN | | | |
| --- | --- | --- | --- | --- | --- | --- | --- | --- | --- |
| Origin | Variable | B | SE | p-value | VIF | B | SE | p-value | VIF |
| **Unadjusted** | | | | | | | | | |
| **SCAPIS** | SUA_50_ | 0.058 | 0.023 | 0.014 | 1.122 | 0.064 | 0.023 | 0.005 | 1.04 |
|  | hs-CRP | 0.105 | 0.034 | 0.002 | 1.122 | 0.174 | 0.036 | <.001 | 1.04 |
| **LitHiR** | SUA_50_ | 0.160 | 0.044 | <.001 | 1.109 | 0.099 | 0.050 | 0.046 | 1.05 |
|  | hs-CRP | 0.184 | 0.074 | 0.014 | 1.109 | 0.077 | 0.086 | 0.377 | 1.05 |
| **The Sagunto** | SUA_50_ | 0.464 | 0.073 | <.001 | 1.062 | 0.009 | 0.066 | 0.896 | 1.02 |
|  | hs-CRP | 0.137 | 0.103 | 0.181 | 1.062 | 0.533 | 0.104 | <.001 | 1.02 |
| **Model 1** | | | | | | | | | |
| **SCAPIS** | SUA_50_ | 0.038 | 0.024 | 0.188 | 1.225 | 0.052 | 0.023 | 0.023 | 1.12 |
|  | hs-CRP | 0.108 | 0.036 | 0.003 | 1.343 | 0.140 | 0.036 | <.001 | 1.12 |
|  | Age | 0.065 | 0.007 | <.001 | 1.015 | 0.083 | 0.007 | <.001 | 1.00 |
|  | BMI | 0.000 | 0.007 | 0.991 | 1.399 | 0.019 | 0.008 | 0.026 | 1.19 |
| **LitHiR** | SUA_50_ | 0.139 | 0.043 | 0.001 | 1.167 | 0.090 | 0.052 | 0.081 | 1.18 |
|  | hs-CRP | 0.172 | 0.075 | 0.023 | 1.269 | 0.075 | 0.087 | 0.391 | 1.10 |
|  | Age | 0.112 | 0.015 | <.001 | 1.012 | 0.049 | 0.019 | 0.011 | 1.06 |
|  | BMI | 0.028 | 0.015 | 0.060 | 1.282 | 0.048 | 0.023 | 0.035 | 1.15 |
| **The Sagunto** | SUA_50_ | 0.043 | 0.058 | 0.458 | 1.286 | -0.022 | 0.051 | 0.672 | 1.09 |
|  | hs-CRP | 0.149 | 0.081 | 0.067 | 1.271 | 0.142 | 0.083 | 0.088 | 1.15 |
|  | Age | 0.106 | 0.006 | <.001 | 1.122 | 0.098 | 0.006 | <.001 | 1.04 |
|  | BMI | 0.086 | 0.017 | <.001 | 1.389 | 0.111 | 0.021 | <.001 | 1.18 |
| **Model 2** | | | | | | | | | |
| **SCAPIS** | SUA_50_ | 0.029 | 0.021 | 0.164 | 1.315 | 0.078 | 0.021 | <.001 | 1.25 |
|  | hs-CRP | 0.038 | 0.032 | 0.229 | 1.374 | 0.097 | 0.032 | 0.003 | 1.13 |
|  | Age | 0.033 | 0.006 | <.001 | 1.107 | 0.069 | 0.006 | <.001 | 1.08 |
|  | BMI | -0.037 | 0.006 | <.001 | 1.523 | -0.017 | 0.008 | 0.025 | 1.27 |
|  | eGFR | -0.004 | 0.002 | 0.063 | 1.131 | 0.007 | 0.002 | 0.007 | 1.18 |
|  | HR | 0.037 | 0.003 | <.001 | 1.048 | 0.042 | 0.003 | <.001 | 1.08 |
|  | PP | 0.053 | 0.003 | <.001 | 1.191 | 0.044 | 0.003 | <.001 | 1.10 |
| **LitHiR** | SUA_50_ | 0.095 | 0.041 | 0.022 | 1.318 | 0.085 | 0.053 | 0.113 | 1.28 |
|  | hs-CRP | 0.061 | 0.071 | 0.387 | 1.324 | 0.046 | 0.086 | 0.593 | 1.12 |
|  | Age | 0.064 | 0.016 | <.001 | 1.337 | 0.057 | 0.019 | 0.004 | 1.17 |
|  | BMI | 0.014 | 0.014 | 0.328 | 1.336 | 0.024 | 0.023 | 0.286 | 1.24 |
|  | eGFR | -0.007 | 0.006 | 0.248 | 1.312 | 0.001 | 0.007 | 0.942 | 1.18 |
|  | HR | 0.041 | 0.006 | <.001 | 1.062 | 0.018 | 0.009 | 0.047 | 1.12 |
|  | PP | 0.041 | 0.005 | <.001 | 1.201 | 0.029 | 0.009 | 0.002 | 1.08 |
| **The Sagunto** | SUA_50_ | 0.039 | 0.062 | 0.526 | 1.573 | -0.019 | 0.050 | 0.698 | 1.18 |
|  | hs-CRP | 0.094 | 0.080 | 0.242 | 1.350 | 0.083 | 0.078 | 0.289 | 1.16 |
|  | Age | 0.087 | 0.008 | <.001 | 2.369 | 0.088 | 0.007 | <.001 | 1.82 |
|  | BMI | 0.078 | 0.016 | <.001 | 1.430 | 0.099 | 0.020 | <.001 | 1.22 |
|  | eGFR | -0.004 | 0.006 | 0.524 | 2.530 | -0.004 | 0.006 | 0.467 | 1.80 |
|  | HR | 0.015 | 0.006 | 0.016 | 1.141 | 0.021 | 0.006 | <.001 | 1.07 |
|  | PP | 0.035 | 0.006 | <.001 | 1.452 | 0.044 | 0.006 | <.001 | 1.09 |
| **Model 3** | | | | | | | | | |
| **SCAPIS** | SUA_50_ | 0.037 | 0.021 | 0.085 | 1.030 | 0.070 | 0.022 | 0.001 | 1.28 |
|  | hs-CRP | 0.038 | 0.032 | 0.238 | 1.070 | 0.089 | 0.062 | 0.007 | 1.15 |
|  | Age | 0.031 | 0.006 | <.001 | 1.116 | 0.069 | 0.243 | <0.001 | 1.13 |
|  | BMI | -0.043 | 0.006 | <.001 | 1.120 | -0.018 | -0.056 | 0.025 | 1.34 |
|  | eGFR | -0.004 | 0.002 | 0.069 | 1.069 | 0.006 | 0.059 | 0.013 | 1.21 |
|  | HR | 0.035 | 0.003 | <.001 | 1.067 | 0.041 | 0.320 | <0.001 | 1.14 |
|  | PP | 0.051 | 0.003 | <.001 | 1.237 | 0.041 | 0.299 | <0.001 | 1.14 |
|  | Diabetes | -0.232 | 0.122 | 0.058 | 1.045 | 0.016 | 0.004 | 0.894 | 1.91 |
|  | Smoking | 0.000 | 0.026 | 0.997 | 1.028 | -0.042 | -0.031 | 0.158 | 1.03 |
|  | FPG | 0.163 | 0.028 | <.001 | 1.156 | 0.036 | 0.043 | 0.144 | 1.87 |
|  | TG | -0.047 | 0.032 | 0.144 | 1.049 | 0.048 | 0.039 | 0.090 | 1.12 |
|  | Antihypertensives, yes | 0.215 | 0.067 | 0.001 | 1.115 | 0.201 | 0.066 | 0.006 | 1.24 |
|  | Lipid-lowering therapy, yes | -0.315 | 0.103 | 0.002 | 1.042 | -0.159 | -0.038 | 0.107 | 1.19 |
| **LitHiR** | SUA_50_ | 0.092 | 0.042 | 0.029 | 1.032 | 0.102 | 0.053 | 0.055 | 1.36 |
|  | hs-CRP | 0.034 | 0.072 | 0.636 | 1.050 | -0.008 | -0.006 | 0.927 | 1.16 |
|  | Age | 0.059 | 0.016 | <.001 | 1.172 | 0.054 | 0.175 | 0.007 | 1.27 |
|  | BMI | 0.007 | 0.014 | 0.608 | 1.093 | 0.015 | 0.042 | 0.514 | 1.29 |
|  | eGFR | -0.005 | 0.006 | 0.418 | 1.082 | 0.002 | 0.019 | 0.766 | 1.25 |
|  | HR | 0.035 | 0.006 | <.001 | 1.074 | 0.008 | 0.059 | 0.347 | 1.18 |
|  | PP | 0.040 | 0.005 | <.001 | 1.215 | 0.024 | 0.158 | 0.009 | 1.11 |
|  | Diabetes | 0.188 | 0.178 | 0.291 | 1.067 | 0.448 | 0.128 | 0.074 | 1.56 |
|  | Smoking | -0.253 | 0.136 | 0.064 | 1.046 | -0.235 | -0.116 | 0.049 | 1.06 |
|  | FPG | 0.063 | 0.047 | 0.179 | 1.088 | 0.027 | 0.037 | 0.636 | 1.85 |
|  | TG | 0.078 | 0.046 | 0.096 | 1.079 | 0.049 | 0.168 | 0.009 | 1.26 |
|  | Antihypertensives, yes | 0.216 | 0.141 | 0.125 | 1.060 | 0.219 | 0.085 | 0.175 | 1.21 |
|  | Lipid-lowering therapy, yes | 0.073 | 0.142 | 0.609 | 1.057 | -0.321 | -0.105 | 0.089 | 1.15 |
| **The Sagunto** | SUA_50_ | 0.041 | 0.065 | 0.532 | 1.021 | -0.026 | -0.056 | 0.644 | 1.30 |
|  | hs-CRP | 0.115 | 0.090 | 0.200 | 1.044 | 0.038 | 0.018 | 0.666 | 1.19 |
|  | Age | 0.086 | 0.010 | <.001 | 1.111 | 0.081 | 0.471 | <0.001 | 2.13 |
|  | BMI | 0.064 | 0.017 | <.001 | 1.095 | 0.098 | 0.182 | <0.001 | 1.31 |
|  | eGFR | -0.001 | 0.006 | 0.929 | 1.076 | -0.004 | -0.031 | 0.531 | 1.80 |
|  | HR | 0.018 | 0.007 | 0.013 | 1.064 | 0.015 | 0.086 | 0.033 | 1.17 |
|  | PP | 0.029 | 0.007 | <.001 | 1.198 | 0.044 | 0.249 | <0.001 | 1.19 |
|  | Diabetes | -0.142 | 0.352 | 0.687 | 1.033 | 0.507 | 0.079 | 0.110 | 1.77 |
|  | Smoking | -0.003 | 0.125 | 0.984 | 1.025 | 0.096 | 0.034 | 0.375 | 1.07 |
|  | FPG | 0.257 | 0.070 | <.001 | 1.068 | 0.132 | 0.078 | 0.102 | 1.66 |
|  | TG | 0.020 | 0.137 | 0.882 | 1.036 | 0.032 | 0.014 | 0.731 | 1.23 |
|  | Antihypertensives, yes | 0.411 | 0.245 | 0.096 | 1.059 | 0.059 | 0.012 | 0.791 | 1.52 |
|  | Lipid-lowering therapy, yes | -0.516 | 0.281 | 0.067 | 1.047 | 0.072 | 0.015 | 0.733 | 1.35 |

SUA_50_ corresponds SUA values per 50 µmol/L increase. hs-CRP was analyzed on the logarithmic scale. A 1-unit increase in ln(hs-CRP) ≈ 2.72-fold increase in hs-CRP

Abbreviations: SUA, serum uric acid; SUAq, quartiles of serum uric acid; hs-CRP, high sensitivity C-reactive protein; BMI, body mass index; eGFR, creatinine based estimated glomerular filtration, EKFC equation used; HR, heart rate; PP, pulse pressure; FPG, fasting plasma glucose; TG, triglycerides.

**Supplemental Table S4** Sensitivity analysis. Linear regression with cfPWVz as the outcome

|  | | WOMEN | | | | MEN | | | |
| --- | --- | --- | --- | --- | --- | --- | --- | --- | --- |
| Origin | Variable | B | SE | p-value | VIF | B | SE | p-value | VIF |
| **Unadjusted** | | | | | | | | | |
| **SCAPIS** | SUA_50_ | 0.043 | 0.017 | 0.014 | 1.122 | 0.047 | 0.017 | 0.005 | 1.036 |
|  | hs-CRP | 0.078 | 0.025 | 0.002 | 1.122 | 0.129 | 0.027 | <0.001 | 1.036 |
| **LitHiR** | SUA_50_ | 0.118 | 0.033 | <0.001 | 1.109 | 0.073 | 0.037 | 0.046 | 1.052 |
|  | hs-CRP | 0.136 | 0.055 | 0.014 | 1.109 | 0.057 | 0.064 | 0.377 | 1.052 |
| **The Sagunto** | SUA_50_ | 0.344 | 0.054 | <0.001 | 1.062 | 0.006 | 0.049 | 0.896 | 1.021 |
|  | hs-CRP | 0.102 | 0.076 | 0.181 | 1.062 | 0.395 | 0.077 | <0.001 | 1.021 |
| **Model 1** | | | | | | | | | |
| **SCAPIS** | SUA_50_ | 0.023 | 0.018 | 0.188 | 1.225 | 0.038 | 0.017 | 0.023 | 1.117 |
|  | hs-CRP | 0.08 | 0.027 | 0.003 | 1.343 | 0.104 | 0.027 | <0.001 | 1.115 |
|  | Age | 0.048 | 0.005 | <0.001 | 1.015 | 0.061 | 0.005 | <0.001 | 1.001 |
|  | BMI | −0.00006 | 0.005 | 0.991 | 1.399 | 0.014 | 0.006 | 0.026 | 1.189 |
| **LitHiR** | SUA_50_ | 0.103 | 0.032 | 0.001 | 1.167 | 0.067 | 0.038 | 0.081 | 1.179 |
|  | hs-CRP | 0.127 | 0.056 | 0.023 | 1.269 | 0.055 | 0.065 | 0.391 | 1.104 |
|  | Age | 0.083 | 0.011 | <0.001 | 1.012 | 0.036 | 0.014 | 0.011 | 1.063 |
|  | BMI | 0.021 | 0.011 | 0.060 | 1.282 | 0.035 | 0.017 | 0.035 | 1.152 |
| **The Sagunto** | SUA_50_ | 0.032 | 0.043 | 0.458 | 1.286 | −0.016 | 0.038 | 0.672 | 1.089 |
|  | hs-CRP | 0.11 | 0.06 | 0.067 | 1.271 | 0.105 | 0.061 | 0.088 | 1.15 |
|  | Age | 0.079 | 0.004 | <0.001 | 1.122 | 0.073 | 0.004 | <0.001 | 1.042 |
|  | BMI | 0.064 | 0.012 | <0.001 | 1.389 | 0.082 | 0.015 | <0.001 | 1.182 |
| **Model 2** | | | | | | | | | |
| **SCAPIS** | SUA_50_ | 0.022 | 0.016 | 0.164 | 1.315 | 0.058 | 0.016 | <0.001 | 1.251 |
|  | hs-CRP | 0.028 | 0.024 | 0.229 | 1.374 | 0.072 | 0.024 | 0.003 | 1.13 |
|  | Age | 0.024 | 0.004 | <0.001 | 1.107 | 0.051 | 0.005 | <0.001 | 1.079 |
|  | BMI | −0.027 | 0.005 | <0.001 | 1.523 | −0.013 | 0.006 | 0.025 | 1.265 |
|  | eGFR | −0.003 | 0.002 | 0.063 | 1.131 | 0.005 | 0.002 | 0.007 | 1.178 |
|  | HR | 0.028 | 0.002 | <0.001 | 1.048 | 0.031 | 0.002 | <0.001 | 1.078 |
|  | PP | 0.039 | 0.002 | <0.001 | 1.191 | 0.033 | 0.002 | <0.001 | 1.104 |
| **LitHiR** | SUA_50_ | 0.07 | 0.031 | 0.022 | 1.318 | 0.063 | 0.04 | 0.113 | 1.277 |
|  | hs-CRP | 0.045 | 0.052 | 0.387 | 1.324 | 0.034 | 0.064 | 0.593 | 1.12 |
|  | Age | 0.048 | 0.012 | <0.001 | 1.337 | 0.042 | 0.014 | 0.004 | 1.166 |
|  | BMI | 0.01 | 0.01 | 0.328 | 1.336 | 0.018 | 0.017 | 0.286 | 1.236 |
|  | eGFR | −0.005 | 0.005 | 0.248 | 1.312 | 0 | 0.005 | 0.942 | 1.184 |
|  | HR | 0.031 | 0.005 | <0.001 | 1.062 | 0.013 | 0.007 | 0.047 | 1.118 |
|  | PP | 0.031 | 0.004 | <0.001 | 1.201 | 0.021 | 0.007 | 0.002 | 1.079 |
| **The Sagunto** | SUA_50_ | 0.029 | 0.046 | 0.526 | 1.573 | −0.014 | 0.037 | 0.698 | 1.179 |
|  | hs-CRP | 0.069 | 0.059 | 0.242 | 1.35 | 0.061 | 0.058 | 0.289 | 1.163 |
|  | Age | 0.065 | 0.006 | <0.001 | 2.369 | 0.065 | 0.005 | <0.001 | 1.823 |
|  | BMI | 0.058 | 0.012 | <0.001 | 1.43 | 0.073 | 0.014 | <0.001 | 1.216 |
|  | eGFR | −0.003 | 0.004 | 0.524 | 2.53 | −0.003 | 0.004 | 0.467 | 1.804 |
|  | HR | 0.011 | 0.005 | 0.016 | 1.141 | 0.016 | 0.004 | <0.001 | 1.07 |
|  | PP | 0.026 | 0.005 | <0.001 | 1.452 | 0.033 | 0.004 | <0.001 | 1.086 |
| **Model 3** | | | | | | | | | |
| **SCAPIS** | SUA_50_ | 0.027 | 0.016 | 0.085 | 1.357 | 0.052 | 0.016 | 0.001 | 1.281 |
|  | hs-CRP | 0.028 | 0.024 | 0.238 | 1.387 | 0.066 | 0.024 | 0.007 | 1.147 |
|  | Age | 0.023 | 0.004 | <0.001 | 1.148 | 0.051 | 0.005 | <0.001 | 1.132 |
|  | BMI | −0.032 | 0.005 | <0.001 | 1.583 | −0.014 | 0.006 | 0.025 | 1.338 |
|  | eGFR | −0.003 | 0.002 | 0.069 | 1.144 | 0.005 | 0.002 | 0.013 | 1.208 |
|  | HR | 0.026 | 0.002 | <0.001 | 1.074 | 0.03 | 0.002 | <0.001 | 1.136 |
|  | PP | 0.038 | 0.002 | <0.001 | 1.25 | 0.031 | 0.002 | <0.001 | 1.136 |
|  | Diabetes | −0.172 | 0.091 | 0.058 | 1.617 | 0.012 | 0.088 | 0.894 | 1.909 |
|  | Smoking | 0 | 0.019 | 0.997 | 1.026 | −0.031 | 0.022 | 0.158 | 1.034 |
|  | FPG | 0.121 | 0.021 | <0.001 | 1.62 | 0.027 | 0.018 | 0.144 | 1.869 |
|  | TG | −0.035 | 0.024 | 0.144 | 1.191 | 0.035 | 0.021 | 0.09 | 1.119 |
|  | Antihypertensives, yes | 0.159 | 0.049 | 0.001 | 1.239 | 0.149 | 0.054 | 0.006 | 1.237 |
|  | Lipid-lowering therapy, yes | −0.233 | 0.076 | 0.002 | 1.169 | −0.118 | 0.073 | 0.107 | 1.189 |
| **LitHiR** | SUA_50_ | 0.068 | 0.031 | 0.029 | 1.387 | 0.076 | 0.039 | 0.055 | 1.356 |
|  | hs-CRP | 0.025 | 0.053 | 0.636 | 1.399 | −0.006 | 0.062 | 0.927 | 1.156 |
|  | Age | 0.044 | 0.012 | <0.001 | 1.392 | 0.04 | 0.015 | 0.007 | 1.271 |
|  | BMI | 0.005 | 0.011 | 0.608 | 1.443 | 0.011 | 0.017 | 0.514 | 1.286 |
|  | eGFR | −0.004 | 0.005 | 0.418 | 1.338 | 0.002 | 0.005 | 0.766 | 1.247 |
|  | HR | 0.026 | 0.005 | <0.001 | 1.165 | 0.006 | 0.007 | 0.347 | 1.182 |
|  | PP | 0.03 | 0.004 | <0.001 | 1.263 | 0.018 | 0.007 | 0.009 | 1.113 |
|  | Diabetes | 0.139 | 0.132 | 0.291 | 1.612 | 0.332 | 0.185 | 0.074 | 1.556 |
|  | Smoking | −0.188 | 0.101 | 0.064 | 1.025 | −0.174 | 0.088 | 0.049 | 1.061 |
|  | FPG | 0.047 | 0.035 | 0.179 | 1.674 | 0.02 | 0.042 | 0.636 | 1.85 |
|  | TG | 0.057 | 0.034 | 0.096 | 1.182 | 0.036 | 0.014 | 0.009 | 1.262 |
|  | Antihypertensives, yes | 0.16 | 0.104 | 0.125 | 1.108 | 0.162 | 0.119 | 0.175 | 1.21 |
|  | Lipid-lowering therapy, yes | 0.054 | 0.105 | 0.609 | 1.084 | −0.238 | 0.14 | 0.089 | 1.152 |
| **The Sagunto** | SUA_50_ | 0.03 | 0.048 | 0.532 | 1.567 | −0.019 | 0.042 | 0.644 | 1.295 |
|  | hs-CRP | 0.086 | 0.067 | 0.2 | 1.429 | 0.028 | 0.064 | 0.666 | 1.189 |
|  | Age | 0.063 | 0.007 | <0.001 | 2.614 | 0.06 | 0.007 | <0.001 | 2.132 |
|  | BMI | 0.047 | 0.013 | <0.001 | 1.449 | 0.072 | 0.017 | <0.001 | 1.309 |
|  | eGFR | 0 | 0.005 | 0.929 | 2.408 | −0.003 | 0.005 | 0.531 | 1.796 |
|  | HR | 0.013 | 0.005 | 0.013 | 1.254 | 0.011 | 0.005 | 0.033 | 1.168 |
|  | PP | 0.022 | 0.005 | <0.001 | 1.468 | 0.032 | 0.005 | <0.001 | 1.189 |
|  | Diabetes | −0.105 | 0.261 | 0.687 | 1.864 | 0.375 | 0.234 | 0.11 | 1.77 |
|  | Smoking | −0.002 | 0.093 | 0.984 | 1.307 | 0.071 | 0.08 | 0.375 | 1.072 |
|  | FPG | 0.19 | 0.052 | <0.001 | 1.808 | 0.098 | 0.06 | 0.102 | 1.658 |
|  | TG | 0.015 | 0.102 | 0.882 | 1.244 | 0.023 | 0.068 | 0.731 | 1.227 |
|  | Antihypertensives, yes | 0.304 | 0.182 | 0.096 | 1.618 | 0.044 | 0.165 | 0.791 | 1.515 |
|  | Lipid-lowering therapy, yes | −0.383 | 0.208 | 0.067 | 1.869 | 0.053 | 0.155 | 0.733 | 1.354 |

SUA_50_ corresponds SUA values per 50 µmol/L increase. hs-CRP was analyzed on the logarithmic scale. A 1-unit increase in ln(hs-CRP) ≈ 2.72-fold increase in hs-CRP

Abbreviations: SUA, serum uric acid; SUAq, quartiles of serum uric acid; hs-CRP, high sensitivity C-reactive protein; BMI, body mass index; eGFR, creatinine based estimated glomerular filtration, EKFC equation used; HR, heart rate; PP, pulse pressure; FPG, fasting plasma glucose; TG, triglycerides.

**Supplemental Table S5** Odds of cfPWV above 10m/s. Logistic regression analysis

|  |  | **WOMEN** | | | | | **MEN** | | | | |
| --- | --- | --- | --- | --- | --- | --- | --- | --- | --- | --- | --- |
| **Origin** | **Predictor** | **B** | **SE** | **p-value** | **OR** | **95% CI for OR** | **B** | **SE** | **p-value** | **OR** | **95% CI for OR** |
| **Unadjusted** | | | | | | | | | | | |
| **SCAPIS** | SUA_50_ | 0.087 | 0.096 | 0.363 | 1.091 | 0.904 – 1.317 | 0.064 | 0.058 | 0.274 | 1.066 | 0.951 – 1.195 |
|  | hs-CRP | 0.139 | 0.139 | 0.317 | 1.149 | 0.875 – 1.509 | 0.167 | 0.088 | 0.057 | 1.182 | 0.995 – 1.403 |
| **LitHiR** | SUA_50_ | 0.241 | 0.079 | 0.002 | 1.272 | 1.089 – 1.487 | 0.061 | 0.164 | 0.711 | 1.063 | 0.770 – 1.466 |
|  | hs-CRP | 0.320 | 0.138 | 0.021 | 1.378 | 1.050 – 1.807 | -0.011 | 0.286 | 0.968 | 0.989 | 0.565 – 1.732 |
| **The Sagunto** | SUA_50_ | 0.242 | 0.074 | 0.001 | 1.274 | 1.102 – 1.473 | 0.035 | 0.071 | 0.625 | 1.035 | 0.900 – 1.191 |
|  | hs-CRP | 0.146 | 0.108 | 0.178 | 1.157 | 0.936 – 1.430 | 0.410 | 0.115 | <.001 | 1.506 | 1.202 – 1.888 |
| **Model 1** | | | | | | | | | | | |
| **SCAPIS** | SUA_50_ | 0.097 | 0.103 | 0.349 | 1.101 | 0.900 – 1.348 | 0.06 | 0.062 | 0.334 | 1.062 | 0.940 – 1.199 |
|  | hs-CRP | 0.279 | 0.148 | 0.059 | 1.321 | 0.989 – 1.764 | 0.133 | 0.095 | 0.161 | 1.142 | 0.948–1.375 |
|  | Age | 0.152 | 0.032 | <.001 | 1.164 | 1.094 – 1.238 | 0.172 | 0.021 | <0.001 | 1.187 | 1.139–1.238 |
|  | BMI | -0.071 | 0.032 | 0.027 | 0.931 | 0.874 – 0.992 | 0.018 | 0.022 | 0.428 | 1.018 | 0.974–1.064 |
|  |  |  |  |  |  |  |  |  |  |  |  |
| **LitHiR** | SUA_50_ | 0.239 | 0.083 | 0.004 | 1.270 | 1.079 – 1.494 | 0.077 | 0.177 | 0.662 | 1.081 | 0.764 – 1.528 |
|  | hsCRP | 0.367 | 0.152 | 0.016 | 1.443 | 1.071 – 1.945 | 0.012 | 0.289 | 0.966 | 1.012 | 0.575–1.783 |
|  | Age | 0.139 | 0.035 | <.001 | 1.150 | 1.074 – 1.230 | 0.077 | 0.065 | 0.237 | 1.080 | 0.951–1.226 |
|  | BMI | 0.010 | 0.030 | 0.747 | 1.010 | 0.952 – 1.071 | 0.025 | 0.076 | 0.745 | 1.025 | 0.884–1.189 |
| **The Sagunto** | SUA_50_ | −0.205 | 0.101 | 0.043 | 0.815 | 0.668 – 0.993 | 0.064 | 0.088 | 0.467 | 1.066 | 0.898 – 1.266 |
|  | hs-CRP | 0.210 | 0.166 | 0.206 | 1.234 | 0.891 – 1.709 | 0.202 | 0.146 | 0.168 | 1.223 | 0.919–1.629 |
|  | Age | 0.138 | 0.015 | <.001 | 1.148 | 1.115 – 1.182 | 0.126 | 0.015 | <0.001 | 1.135 | 1.102–1.168 |
|  | BMI | 0.124 | 0.031 | <.001 | 1.132 | 1.064 – 1.204 | 0.131 | 0.036 | <0.001 | 1.139 | 1.062–1.223 |
| **Model 2** | | | | | | | | | | | |
| **SCAPIS** | SUA_50_ | 0.075 | 0.117 | 0.52 | 1.078 | 0.858 – 1.355 | 0.145 | 0.068 | 0.034 | 1.156 | 1.011 – 1.322 |
|  | hs-CRP | 0.163 | 0.170 | 0.338 | 1.177 | 0.844 – 1.642 | 0.099 | 0.102 | 0.335 | 1.104 | 0.903 – 1.349 |
|  | Age | 0.071 | 0.035 | 0.044 | 1.074 | 1.002 – 1.151 | 0.165 | 0.024 | <.001 | 1.180 | 1.126 – 1.236 |
|  | BMI | -0.153 | 0.037 | <.001 | 0.858 | 0.798 – 0.922 | -0.050 | 0.024 | 0.039 | 0.951 | 0.907 – 0.998 |
|  | eGFR | -0.014 | 0.012 | 0.214 | 0.986 | 0.964 – 1.008 | 0.019 | 0.008 | 0.017 | 1.019 | 1.003 – 1.035 |
|  | HR | 0.081 | 0.014 | <.001 | 1.084 | 1.054 – 1.114 | 0.075 | 0.009 | <.001 | 1.078 | 1.059 – 1.097 |
|  | PP | 0.119 | 0.012 | <.001 | 1.126 | 1.100 – 1.154 | 0.082 | 0.009 | <.001 | 1.085 | 1.065 – 1.105 |
| **LitHiR** | SUA_50_ | 0.195 | 0.098 | 0.047 | 1.216 | 1.003 – 1.474 | −0.035 | 0.192 | 0.853 | 0.965 | 0.663 – 1.406 |
|  | hs-CRP | 0.173 | 0.171 | 0.310 | 1.189 | 0.851 – 1.661 | -0.044 | 0.303 | 0.885 | 0.957 | 0.528 – 1.735 |
|  | Age | 0.070 | 0.041 | 0.084 | 1.073 | 0.991 – 1.162 | 0.077 | 0.071 | 0.275 | 1.080 | 0.940 – 1.241 |
|  | BMI | -0.024 | 0.033 | 0.465 | 0.976 | 0.915 – 1.041 | -0.008 | 0.080 | 0.923 | 0.992 | 0.848 – 1.161 |
|  | eGFR | -0.015 | 0.015 | 0.306 | 0.985 | 0.957 – 1.014 | -0.008 | 0.025 | 0.766 | 0.993 | 0.945 – 1.043 |
|  | HR | 0.064 | 0.014 | <.001 | 1.066 | 1.037 – 1.097 | 0.029 | 0.032 | 0.369 | 1.030 | 0.966 – 1.097 |
|  | PP | 0.053 | 0.013 | <.001 | 1.054 | 1.029 – 1.081 | 0.037 | 0.030 | 0.223 | 1.038 | 0.978 – 1.102 |
| **The Sagunto** | SUA_50_ | −0.192 | 0.12 | 0.109 | 0.825 | 0.652 – 1.044 | 0.040 | 0.1 | 0.691 | 1.04 | 0.855 – 1.266 |
|  | hs-CRP | 0.116 | 0.175 | 0.507 | 1.123 | 0.797 – 1.584 | 0.079 | 0.161 | 0.622 | 1.083 | 0.790 – 1.484 |
|  | Age | 0.124 | 0.019 | <.001 | 1.132 | 1.090 – 1.175 | 0.102 | 0.018 | <.001 | 1.108 | 1.069 – 1.148 |
|  | BMI | 0.121 | 0.032 | <.001 | 1.129 | 1.060 – 1.203 | 0.127 | 0.039 | 0.001 | 1.135 | 1.051 – 1.226 |
|  | eGFR | -0.002 | 0.012 | 0.875 | 0.998 | 0.974 – 1.022 | -0.008 | 0.011 | 0.463 | 0.992 | 0.971 – 1.013 |
|  | HR | 0.021 | 0.013 | 0.095 | 1.021 | 0.996 – 1.047 | 0.027 | 0.012 | 0.030 | 1.027 | 1.003 – 1.052 |
|  | PP | 0.031 | 0.011 | 0.007 | 1.031 | 1.008 – 1.055 | 0.077 | 0.014 | <.001 | 1.080 | 1.051 – 1.111 |
| **Model 3** | | | | | | | | | | | |
| **SCAPIS** | SUA_50_ | 0.143 | 0.123 | 0.247 | 1.153 | 0.906 – 1.468 | 0.144 | 0.071 | 0.041 | 1.155 | 1.006 – 1.327 |
|  | hs-CRP | 0.155 | 0.177 | 0.380 | 1.168 | 0.826 – 1.651 | 0.066 | 0.107 | 0.540 | 1.068 | 0.866 – 1.317 |
|  | Age | 0.083 | 0.037 | 0.024 | 1.086 | 1.011 – 1.167 | 0.174 | 0.025 | <0.001 | 1.190 | 1.133 – 1.249 |
|  | BMI | -0.162 | 0.039 | <.001 | 0.850 | 0.787 – 0.919 | -0.056 | 0.027 | 0.037 | 0.946 | 0.897 – 0.997 |
|  | eGFR | -0.012 | 0.012 | 0.322 | 0.988 | 0.966 – 1.012 | 0.017 | 0.008 | 0.046 | 1.017 | 1.000 – 1.034 |
|  | HR | 0.077 | 0.015 | <.001 | 1.080 | 1.049 – 1.111 | 0.069 | 0.009 | <0.001 | 1.072 | 1.052 – 1.092 |
|  | PP | 0.125 | 0.013 | <.001 | 1.133 | 1.104 – 1.163 | 0.078 | 0.010 | <0.001 | 1.081 | 1.061 – 1.102 |
|  | Diabetes | -0.318 | 0.582 | 0.585 | 0.728 | 0.232 – 2.277 | 0.091 | 0.328 | 0.782 | 1.095 | 0.576 – 2.082 |
|  | Smoking | -0.150 | 0.157 | 0.339 | 0.861 | 0.633 – 1.170 | -0.093 | 0.102 | 0.362 | 0.911 | 0.745 – 1.113 |
|  | FPG | 0.269 | 0.118 | 0.022 | 1.309 | 1.039 – 1.648 | 0.088 | 0.062 | 0.153 | 1.092 | 0.968 – 1.232 |
|  | TG | -0.227 | 0.238 | 0.339 | 0.797 | 0.500 – 1.270 | 0.159 | 0.083 | 0.055 | 1.172 | 0.996 – 1.379 |
|  | Antihypertensives, yes | -0.384 | 0.348 | 0.271 | 0.681 | 0.344 – 1.349 | 0.086 | 0.221 | 0.698 | 1.090 | 0.706 – 1.682 |
|  | Lipid-lowering therapy, yes | -1.570 | 0.740 | 0.034 | 0.208 | 0.049 – 0.887 | -0.421 | 0.321 | 0.189 | 0.656 | 0.350 – 1.230 |
| **LitHiR** | SUA_50_ | 0.186 | 0.101 | 0.065 | 1.205 | 0.989– 1.468 | -0.016 | 0.196 | 0.936 | 0.984 | 0.671 – 1.445 |
|  | hs-CRP | 0.194 | 0.175 | 0.267 | 1.214 | 0.862 – 1.711 | -0.176 | 0.309 | 0.568 | 0.838 | 0.458 – 1.535 |
|  | Age | 0.058 | 0.041 | 0.163 | 1.059 | 0.977 – 1.149 | 0.070 | 0.075 | 0.352 | 1.072 | 0.926 – 1.243 |
|  | BMI | -0.031 | 0.035 | 0.383 | 0.970 | 0.905 – 1.039 | -0.025 | 0.082 | 0.764 | 0.976 | 0.831 – 1.145 |
|  | eGFR | -0.016 | 0.015 | 0.288 | 0.984 | 0.955 – 1.014 | -0.008 | 0.027 | 0.766 | 0.992 | 0.942 – 1.045 |
|  | HR | 0.063 | 0.015 | <.001 | 1.065 | 1.034 – 1.098 | 0.009 | 0.034 | 0.794 | 1.009 | 0.944 – 1.078 |
|  | PP | 0.056 | 0.013 | <.001 | 1.058 | 1.031 – 1.085 | 0.026 | 0.034 | 0.439 | 1.026 | 0.961 – 1.096 |
|  | Diabetes | 0.499 | 0.379 | 0.187 | 1.648 | 0.784 – 3.462 | 0.847 | 0.781 | 0.279 | 2.332 | 0.504 – 10.786 |
|  | Smoking | -1.157 | 0.477 | 0.015 | 0.315 | 0.124 – 0.801 | -0.191 | 0.494 | 0.699 | 0.826 | 0.314 – 2.176 |
|  | FPG | -0.066 | 0.104 | 0.525 | 0.936 | 0.764 – 1.147 | -0.024 | 0.172 | 0.890 | 0.976 | 0.697 – 1.368 |
|  | TG | 0.082 | 0.093 | 0.378 | 1.085 | 0.905 – 1.302 | 0.066 | 0.047 | 0.159 | 1.068 | 0.974 – 1.172 |
|  | Antihypertensives, yes | 0.217 | 0.384 | 0.571 | 1.243 | 0.586 – 2.637 | 0.488 | 0.625 | 0.435 | 1.629 | 0.478 – 5.545 |
|  | Lipid-lowering therapy, yes | 0.315 | 0.337 | 0.350 | 1.370 | 0.708 – 2.653 | -0.354 | 0.716 | 0.621 | 0.702 | 0.173 – 2.856 |
| **The Sagunto** | SUA_50_ | -0.267 | 0.146 | 0.067 | 0.766 | 0.575– 1.019 | 0.059 | 0.106 | 0.580 | 1.060 | 0.861 – 1.306 |
|  | hs-CRP | 0.122 | 0.215 | 0.569 | 1.130 | 0.742 – 1.721 | 0.104 | 0.175 | 0.550 | 1.110 | 0.788 – 1.563 |
|  | Age | 0.119 | 0.026 | <.001 | 1.127 | 1.072 – 1.185 | 0.098 | 0.022 | <0.001 | 1.103 | 1.056 – 1.152 |
|  | BMI | 0.133 | 0.041 | 0.001 | 1.142 | 1.055 – 1.237 | 0.124 | 0.044 | 0.005 | 1.132 | 1.037 – 1.235 |
|  | eGFR | 0.001 | 0.015 | 0.939 | 1.001 | 0.973 – 1.030 | -0.008 | 0.012 | 0.501 | 0.992 | 0.968 – 1.016 |
|  | HR | 0.013 | 0.017 | 0.439 | 1.013 | 0.981 – 1.046 | 0.020 | 0.014 | 0.152 | 1.020 | 0.993 – 1.049 |
|  | PP | 0.030 | 0.014 | 0.040 | 1.030 | 1.001 – 1.060 | 0.068 | 0.016 | <0.001 | 1.070 | 1.038 – 1.104 |
|  | Diabetes | -0.575 | 0.650 | 0.376 | 0.563 | 0.158 – 2.011 | -0.356 | 0.554 | 0.520 | 0.700 | 0.236 – 2.075 |
|  | Smoking | -0.286 | 0.289 | 0.322 | 0.752 | 0.427 – 1.323 | 0.070 | 0.214 | 0.745 | 1.072 | 0.705 – 1.632 |
|  | FPG | 0.138 | 0.160 | 0.387 | 1.149 | 0.839 – 1.571 | 0.280 | 0.142 | 0.049 | 1.323 | 1.001 – 1.747 |
|  | TG | 0.226 | 0.295 | 0.444 | 1.253 | 0.703 – 2.233 | 0.089 | 0.187 | 0.635 | 1.093 | 0.757 – 1.578 |
|  | Antihypertensives, yes | 0.829 | 0.510 | 0.104 | 2.291 | 0.843 – 6.223 | -0.116 | 0.412 | 0.777 | 0.890 | 0.397 – 1.994 |
|  | Lipid-lowering therapy, yes | -0.667 | 0.551 | 0.226 | 0.513 | 0.174 – 1.512 | 0.392 | 0.389 | 0.313 | 1.480 | 0.691 – 3.171 |

SUA_50_ corresponds SUA values per 50 µmol/L increase. hs-CRP was analyzed on the logarithmic scale. A 1-unit increase in ln(hs-CRP) ≈ 2.72-fold increase in hs-CRP

Abbreviations: SUA, serum uric acid; SUAq, quartiles of serum uric acid; Ln(hs-CRP), logarithmic values of high sensitivity C-reactive protein; BMI, body mass index; eGFR, creatinine based estimated glomerular filtration, EKFC equation used; HR, heart rate; PP, pulse pressure; FPG, fasting plasma glucose; TG, triglycerides.

**Supplemental Figure 1** Distribution of age, anthropometrics, hs-CRP and hemodynamic characteristics in sex-specific serum uric acid quartiles.


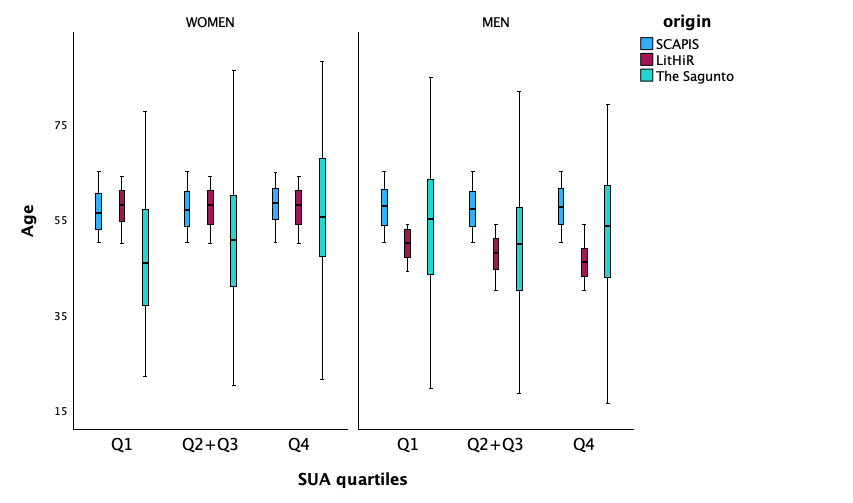

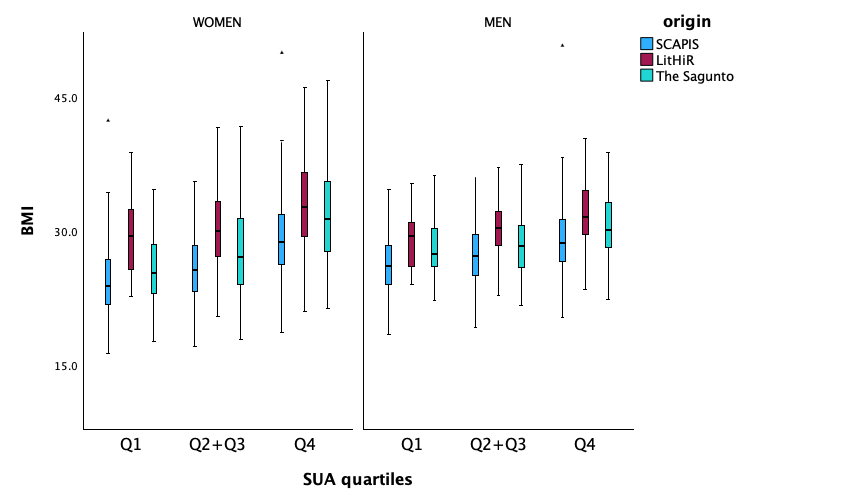

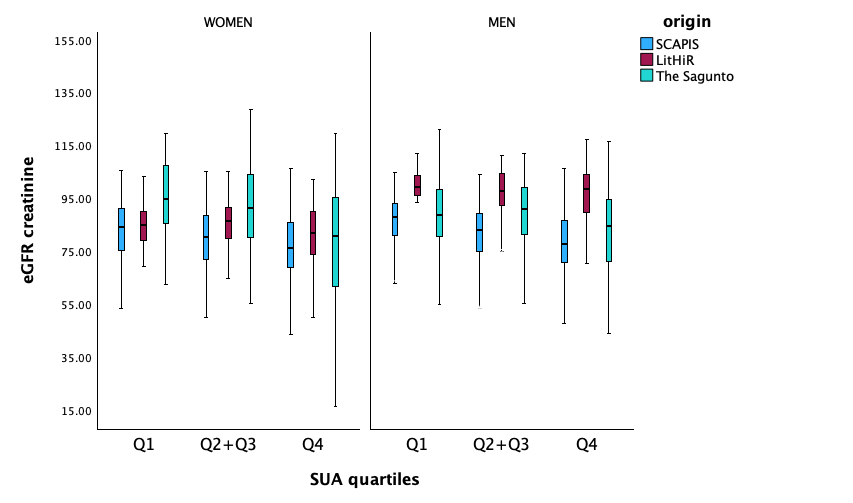

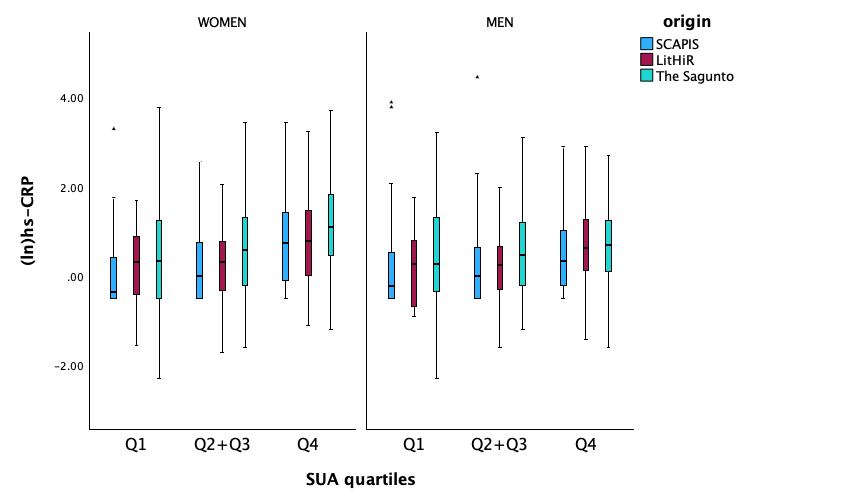

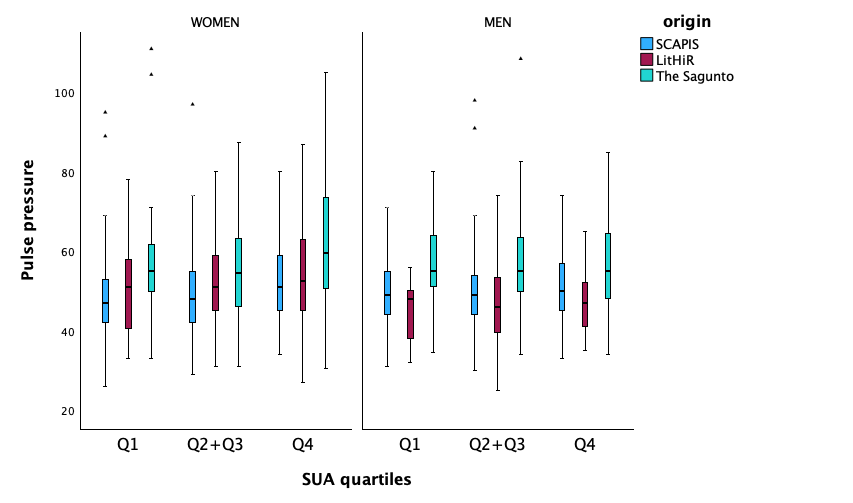

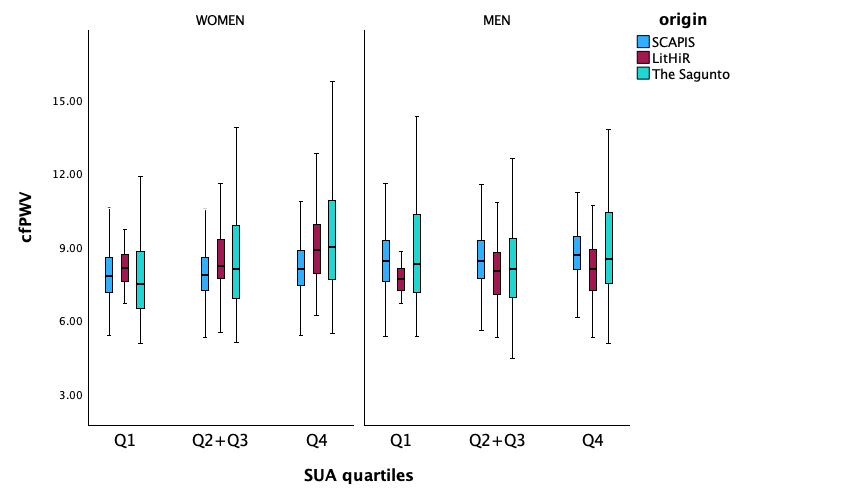

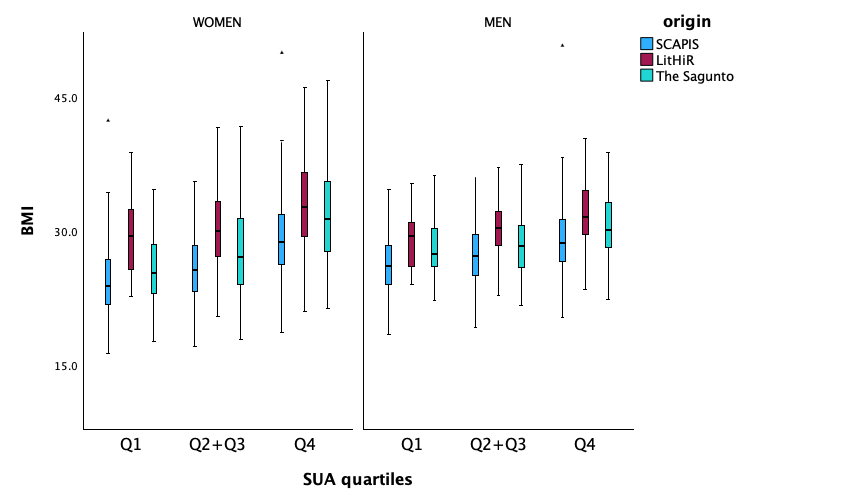


Supplemental Figure 1. In men, cfPWV did not differ across SUA quartiles, except for a near “U”-shaped pattern observed in Spaniards (p < 0.001). hs-CRP exhibited a more complex distribution, increasing across SUA quartiles in Swedish, Lithuanian, and Spanish women, with a less consistent pattern in men. Younger Spanish (p = 0.026) and Lithuanian men (p < 0.001), as well as older Spanish and Swedish women, were more frequently in the highest SUA quartile (p < 0.001 for both groups). Age did not differ across SUA quartiles in Lithuanian women (p = 0.840) or Swedish men (p = 0.669). BMI increased progressively across SUA quartiles (p < 0.001), while pulse pressure showed a right-skewed distribution (all p < 0.001). Kidney function, assessed by creatinine-based eGFR, was lower in the highest SUA quartile across countries and sexes (all p < 0.001; p = 0.002 in Spanish men), except in Lithuanian men (p = 0.315). Data from Sweden are shown in blue, Lithuania in red, and Spain in cyan. The lowest quartile – Q1, the middle quartiles – Q2+Q3, and the highest quartile – Q4.
